# Expansion of Cancer Risk Profile for *BRCA1* and *BRCA2* Pathogenic Variants

**DOI:** 10.1001/jamaoncol.2022.0476

**Published:** 2022-04-14

**Authors:** Yukihide Momozawa, Rumi Sasai, Yoshiaki Usui, Kouya Shiraishi, Yusuke Iwasaki, Yukari Taniyama, Michael T. Parsons, Keijiro Mizukami, Yuya Sekine, Makoto Hirata, Yoichiro Kamatani, Mikiko Endo, Chihiro Inai, Sadaaki Takata, Hidemi Ito, Takashi Kohno, Koichi Matsuda, Seigo Nakamura, Kokichi Sugano, Teruhiko Yoshida, Hidewaki Nakagawa, Keitaro Matsuo, Yoshinori Murakami, Amanda B. Spurdle, Michiaki Kubo

**Affiliations:** 1 Laboratory for Genotyping Development, RIKEN Center for Integrative Medical Sciences, Yokohama, Japan; 2 Division of Cancer Information and Control, Department of Preventive Medicine, Aichi Cancer Center, Nagoya, Japan; 3 Department of Hematology, Oncology and Respiratory Medicine, Okayama University Graduate School of Medicine, Dentistry and Pharmaceuticals Sciences, Okayama, Japan; 4 Division of Genome Biology, National Cancer Center Research Institute, Tokyo, Japan; 5 Division of Genetics and Population Health, QIMR Berghofer Medical Research Institute, Brisbane, Queensland, Australia; 6 Department of Urology, Akita University Graduate School of Medicine, Akita, Japan; 7 Department of Genetic Medicine and Services, National Cancer Center Hospital, Tokyo, Japan; 8 Institute of Medical Science, Division of Molecular Pathology, Department of Cancer Biology, The University of Tokyo, Tokyo, Japan; 9 Laboratory of Complex Trait Genomics, Department of Computational Biology and Medical Sciences, Graduate School of Frontier Sciences, The University of Tokyo, Tokyo, Japan; 10 Division of Descriptive Cancer Epidemiology, Nagoya University Graduate School of Medicine, Nagoya, Japan; 11 Laboratory of Clinical Genome Sequencing, Department of Computational Biology and Medical Sciences, Graduate School of Frontier Sciences, The University of Tokyo, Tokyo, Japan; 12 Division of Breast Surgical Oncology, Department of Surgery, Showa University School of Medicine, Tokyo, Japan; 13 Department of Genetic Medicine, Kyoundo Hospital, Sasaki Foundation, Tokyo, Japan; 14 Laboratory for Cancer Genomics, RIKEN Center for Integrative Medical Sciences, Yokohama, Japan; 15 Division of Cancer Epidemiology and Prevention, Department of Preventive Medicine, Aichi Cancer Center, Nagoya, Japan; 16 Division of Cancer Epidemiology, Nagoya University Graduate School of Medicine, Nagoya, Japan; 17 RIKEN Center for Integrative Medical Sciences, Yokohama, Japan

## Abstract

**Importance:**

The clinical importance of genetic testing of *BRCA1* and *BRCA2* in breast, ovarian, prostate, and pancreatic cancers is widely recognized. However, there is insufficient evidence to include other cancer types that are potentially associated with *BRCA1* and *BRCA2* in clinical management guidelines.

**Objective:**

To evaluate the association of *BRCA1 *and *BRCA2* pathogenic variants with additional cancer types and their clinical characteristics in 100 914 individuals across 14 cancer types.

**Design, Setting, and Participants:**

This case-control analysis to identify cancer types and clinical characteristics associated with pathogenic variants in *BRCA1* and *BRCA2* included DNA samples and clinical information from 63 828 patients with 14 common cancer types and 37 086 controls that were sourced from a multi-institutional hospital-based registry, BioBank Japan, between April 2003 and March 2018. The data were analyzed between August 2019 and October 2021.

**Main Outcomes and Measures:**

Germline pathogenic variants in coding regions and 2 bp flanking intronic sequences in *BRCA1* and *BRCA2* were identified by a multiplex polymerase chain reaction–based target sequence method. Associations of (likely) pathogenic variants with each cancer type were assessed by comparing pathogenic variant carrier frequency between patients in each cancer type and controls.

**Results:**

A total of 65 108 patients (mean [SD] age at diagnosis, 64.1 [11.6] years; 27 531 [42.3%] female) and 38 153 controls (mean [SD] age at registration, 61.8 [14.6] years; 17 911 [46.9%] female) were included in this study. A total of 315 unique pathogenic variants were identified. Pathogenic variants were associated with *P* < 1 × 10^−4^ with an odds ratio (OR) of greater than 4.0 in biliary tract cancer (OR, 17.4; 95% CI, 5.8-51.9) in *BRCA1*, esophageal cancer (OR, 5.6; 95% CI, 2.9-11.0) in *BRCA2*, and gastric cancer (OR, 5.2; 95% CI, 2.6-10.5) in *BRCA1*, and (OR, 4.7; 95% CI, 3.1-7.1) in *BRCA2* in addition to the 4 established cancer types. We also observed an association with 2 and 4 other cancer types in *BRCA1* and *BRCA2*, respectively. Biliary tract, female breast, ovarian, and prostate cancers showed enrichment of carrier patients according to the increased number of reported cancer types in relatives.

**Conclusions and Relevance:**

The results of this large-scale registry-based case-control study suggest that pathogenic variants in *BRCA1 *and *BRCA2 *were associated with the risk of 7 cancer types. These results indicate broader clinical relevance of *BRCA1 *and *BRCA2* genetic testing.

## Key Points

**Question:**

Which cancer types and their clinical characteristics are associated with pathogenic variants in *BRCA1* and *BRCA2* in addition to breast, ovarian, prostate, and pancreatic cancers?

**Findings:**

In this case-control study of 63 828 patients with 14 common cancer types and 37 086 controls, pathogenic variants in *BRCA1* were associated with biliary tract cancer, in *BRCA2* with esophageal cancer, and in *BRCA1/2* with gastric cancer.

**Meaning:**

The study results suggest that the range of cancer types associated with pathogenic variants in *BRCA1 *and* BRCA2* is broader than that determined from previous analyses, potentially indicating the broader clinical relevance of *BRCA1/2* genetic testing.

## Teaser

This case-control study examines the association of *BRCA1 *and *BRCA2* pathogenic variants with additional cancer types and their clinical characteristics in 100 914 Japanese individuals across 14 cancer types.

## Introduction

*BRCA1* and *BRCA2* were identified in the 1990s as the causative genes underlying hereditary breast and ovarian cancer syndrome.^1^ Genetic testing began during the same decade for treatment of patients and their relatives. In addition, polyadenosine diphosphate-ribose polymerase (PARP) inhibitors were developed based on the mechanism of the homologous recombination repair defects associated with pathogenic variants in these genes.^2^ The target *BRCA1 *and *BRCA2* cancer types have expanded to prostate^3^ and pancreatic cancers^4^ because pathogenic variants were enriched in these patients, and the therapeutic efficacy of PARP inhibitors in these cancers has also been shown.^5,6^

Risk for additional cancer types, such as biliary tract cancer,^7^ cervical cancer,^8,9^ colorectal cancer,^9^ endometrial cancer,^9^ esophageal cancer,^10,11^ and stomach cancer,^7,8,11,12,13^ has been reported by analyzing family members for the presence of pathogenic variants and performing case-control analyses. However, the evidence for an association with these cancer types has not been considered sufficient to be adopted into clinical management guidelines, probably because of the small sample size for each cancer type, weak statistical evidence, or a singular focus on family members with pathogenic variants. In addition, evidence to date for different cancer types has been derived from various studies of different design. Robust evidence for additional cancer types in a single population is necessary to design and implement clinical trials that assess efficacy of PARP inhibitors.

We performed a large-scale sequencing study across 14 common cancer types in 63 828 patients and 37 086 controls whose data were drawn from a Japanese nationwide biobank. We used these data to estimate the risk of each cancer type and clinical characteristics associated with pathogenic variant carrier status. These data provide a broad view of cancer risks associated with pathogenic variants in *BRCA1 *and *BRCA2*.

## Methods

### Participants

An overall procedure is shown in eFigure 1 in the Supplement. We obtained samples from 65 108 patients with 14 cancer different types (biliary tract, breast, cervical, colorectal, endometrial, esophageal, gastric, liver, lung, lymphoma, ovarian, pancreatic, prostate, and kidney) from BioBank Japan, a multi-institutional, hospital-based registry that collected DNA and clinical information from across Japan between April 2003 and March 2018.^14,15^ Family history of cancer refers to reported cancer in first-degree and/or second-degree relatives. Among them, 4128 patients (6.3%) had 2 to 5 cancer types. We also enrolled 38 153 controls 20 years or older with no history or family history of cancers. Compared with our previous publications for breast,^16^ colorectal,^17^ pancreatic,^18^ and prostate^19^ cancers, the analyses presented included 14 448 additional controls and 8247 additional cancer cases (2984 breast [36.2%], 3722 colorectal [45.1%], 1535 pancreatic [18.6%], and 6 prostate [0.1%]).

All participants provided written informed consent. The study was approved by the ethical committees of the Institute of Medical Sciences, University of Tokyo, and RIKEN Center for Integrative Medical Sciences.

### Sequencing and Bioinformatics

For germline sequencing, we analyzed all coding regions and 2 bp flanking intronic sequences (16 111 bp) of all transcripts of *BRCA1* (CCDS11453-6, 9) and *BRCA2* (CCDS9344) that were registered in the Consensus CDS, release 15,^20^ by a multiplex polymerase chain reaction–based target sequence method.^21^ After sequencing the pooled DNA libraries using 2 × 150-bp paired-end reads on a HiSeq2500 (Illumina), the genetic variants were identified using the GATK (version 3.7-0; Broad Institute).^22^ We deposited custom scripts at https://github.com/Laboratory-for-Genotyping-Development/TargetSequence.git.

We determined the association of genetic variants with the amino acid sequence using the SnpEff, version 4.3t.^23^ Protein position was reported according to CCDS11456 (the longest one) for *BRCA1* and CCDS9344 for *BRCA2*. We assigned clinical significance for all variants using *BRCA1 *and *BRCA2* variant classification criteria that were developed by members of the Evidence-based Network for the Interpretation of Germline Mutant Alleles Consortium.^24,25^ Pathogenic and likely pathogenic variants were collectively referred to as pathogenic variants.

### Statistical Analysis

Because sex differences in the contribution of pathogenic variants to breast cancer are well known,^26^ we analyzed females and males separately for breast cancer. We conducted 2 separate association analyses. A logistic regression analysis under a dominant model with age at diagnosis for cases and age at registration for controls as a covariate was used. We eliminated samples without age at diagnosis or registration from this calculation. The first analysis examined all patients and controls for each cancer type. Including the control participants without family history would improve the power to detect association but lead to biased risk estimates. Therefore, we reported the results of association testing without risk estimates. For the second analysis, we calculated cancer risk using selected patients with cancer without a family history for comparison with controls without a family history to minimize overestimation of odds ratios (ORs). The cumulative risk and its 95% CIs of each cancer to age 85 years were estimated for carriers and noncarriers of pathogenic variants in *BRCA1* and *BRCA2* using the method described by previous studies^27,28,29,30^ (eAppendix in the Supplement).

We also investigated how family history is useful in detecting patients with pathogenic variants. Family history of a given cancer type was denoted if at least 1 relative reported that cancer type. In each of 7 associated cancer types identified in this study, we calculated the proportion of patients with pathogenic variants according to reported family history of the 7 associated cancer types.

Statistics methods are described where results are shown. All statistical tests were 2-sided, and statistical significance was set at *P* < .05. The Bonferroni correction was applied to adjust for multiple comparisons. We set the threshold of significance to 1 × 10^−4^ for the burden test based on the justified recommendation from a review article that focused on clinical validity of gene panel sequence tests.^31^ Analyses were performed using R, version 3.5.2 (R Foundation), or Stata, version 16.0 (StataCorp).

## Results

### Participant Characteristics

Table 1 shows the characteristics of 65 108 patients with cancer (representing 69 550 case diagnoses) and 38 153 controls. The mean (SD) age at diagnosis varied between cancer types from 49.7 (13.2) years for cervical cancer to 70.2 (7.3) years for prostate cancer. The proportion of cases reporting a family history of the same cancer type was lowest for endometrial cancer (46 [2.4%]) and highest for gastric cancer (3010 [28.1%]).

**Table 1.** Demographic and Clinical Characteristics of the Participants

Cancer type	No. of samples			Age at diagnosis, mean (SD), y	Family history of the same cancer type, %^a^
	Female	Male	Total		
Case					
Biliary tract	320	567	887	68.8 (9.5)	2.5
Breast	10 021	67	10 088	56.4 (12.1)	11.3
Cervical	1923	0	1923	49.7 (13.2)	3.1
Colorectal	5278	8759	14 037	65.5 (10.7)	14.4
Endometrial	1957	0	1957	56.3 (10.9)	2.4
Esophageal	337	1957	2294	65.2 (8.5)	6.1
Gastric	2733	7972	10 705	65.7 (10.5)	28.1
Liver	957	2830	3787	67.3 (9.4)	10.0
Lung	2590	4873	7463	66.5 (9.6)	14.4
Lymphoma	743	956	1699	60.2 (14.0)	2.4
Ovarian	1492	0	1492	53.7 (12.2)	3.8
Pancreatic	384	627	1011	67.2 (9.9)	7.8
Prostate	0	11 358	11 358	70.2 (7.3)	8.2
Kidney	218	631	849	61.3 (11.2)	2.6
Total					
Patients	27 531	37 577	65 108	NA	13.9
Cases^b^	28 953	40 597	69 550	64.1 (11.6)	13.0
Control	17 911	20 242	38 153	61.8 (14.6)^c^	NA
Total	45 442	57 819	103 261	NA	NA

Abbreviation: NA, not applicable.

^a^ Proportion of patients with relatives who had the same cancer types.

^b^ Because 4128 patients (6.3%) had more than 1 cancer type, the numbers of patients and cases are described separately.

^c^ Age at registration was used for the control group.

### Pathogenic Variants

After quality control, 63 828 patients (68 219 case diagnoses) and 37 086 controls were included, with 99.85% of the target region covered by at least 20 sequence reads. We applied 1810 genetic variants to the interpretation of clinical significance. After standardized review, 315 variants (17.4%) were assigned as pathogenic (eTable 1 in the Supplement). eFigure 2 in the Supplement shows the distribution of pathogenic variants found in patients. Three *BRCA1* and 8 *BRCA2* pathogenic variants were observed in 10 or more patients.

The proportion of pathogenic variants in cases differed significantly across the 7 regions serviced by hospitals in Japan by *χ^2^* test (eFigure 3 in the Supplement). There was a 5.8-fold difference in *BRCA1* between 0.15% in Tokai-Hokuriku and 0.85% in Tohoku. *BRCA2* showed a 5.0-fold difference between 0.28% in Okinawa and 1.37% in Kinki. These differences could be largely explained by the different proportion of founder pathogenic variants (eFigure 3 in the Supplement). After excluding these founder pathogenic variants, there was no difference in frequency of *BRCA1* or *BRCA2* pathogenic variants across the 7 regions.

### Carrier Frequency and Disease Risk of Each Cancer Type

**Figure 1.**
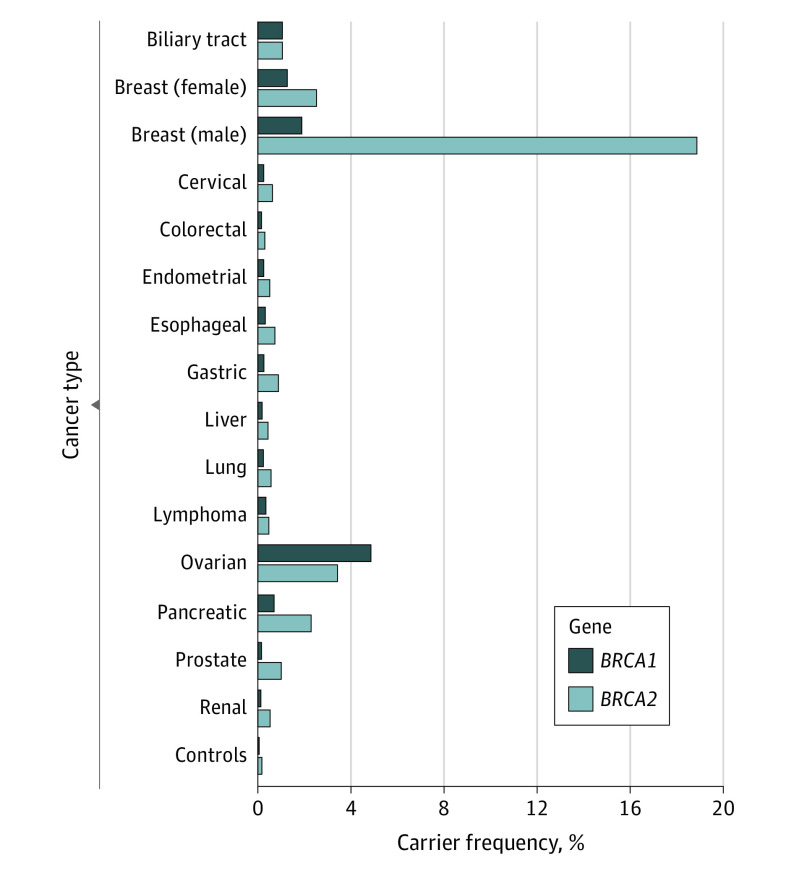
Frequency of Pathogenic Variants in *BRCA1* and *BRCA2* Across 14 Cancer Types and Controls Because sex differences in the contribution of pathogenic variants to breast cancer are well known, carrier frequencies in female and male patients were separately described.

Figure 1 shows the patient carrier frequency for each of the 14 cancer types. Male patients with breast cancer had a very high carrier frequency of pathogenic variants^26^ in *BRCA2* (18.9%), but not *BRCA1* (1.89%). Patients with ovarian cancer showed the next highest proportion (*BRCA1*: 4.86%; *BRCA2*: 3.42%). Frequency exceeding 1% was seen for several other cancer types (2 cancer types for *BRCA1*, 4 cancer types for *BRCA2*). Carrier frequency of pathogenic variants in *BRCA1* was 0.44% in 1 cancer type, 0.85% in 2 cancer types, and 0.69% in 3 cancer types. They were 0.97%, 1.40%, and 1.74% in *BRCA2* (eFigure 4 in the Supplement). Carrier frequency for females and males in each cancer type was significantly correlated for *BRCA1* and *BRCA2* (eFigure 5 in the Supplement).

We performed association analyses with all patients and controls for each cancer type (eTable 2 in the Supplement). Among the 30 analyses, 17 yielded statistically significant results. To provide a more conservative estimate that accounted for control selection criteria, we calculated the disease risk for each cancer type (Table 2); association analyses were not possible for some cancer types because of limited numbers of cases in those subsets. Pathogenic variants in *BRCA1* were significantly associated with increased risk of 5 cancer types: ovarian, female breast, biliary tract, gastric, and pancreatic cancers. Pathogenic variants in *BRCA2* were associated with increased risk of 7 cancer types: female breast, gastric, ovarian, male breast, pancreatic, prostate, and esophageal cancers. These associations were more strongly observed in the analysis with all patients (eTable 2 in the Supplement). We conducted 2 sensitivity analyses in breast cancer. We performed a logistic regression analysis with 7 regions of Japan as a categorical covariate to assess the possibility that population stratification might skew risk estimation. Results for *BRCA1 *and *BRCA2 *(eTable 3 in the Supplement) were comparable with estimates from the main analysis shown in Table 2. We also performed an association analysis with patients with breast cancer only because of a potential bias caused by the presence of more than 1 cancer type. The results for *BRCA1 *and *BRCA2* (eTable 3 in the Supplement) were comparable with those from the main analysis. Heterogeneity of ORs between *BRCA1* and *BRCA2 *were shown in ovarian (*I*^2^, 90.9%) and prostate cancer (*I*^2^, 77.4%) (Table 2).^32^ We also observed an association with lymphoma and lung cancer for *BRCA1* and endometrial, cervical, kidney, and liver cancers for *BRCA2* (Table 2).

**Table 2.** Associations Between Pathogenic Variants in *BRCA1* and *BRCA2* and Risk of the 14 Cancer Types

Cancer type	*BRCA1*		*BRCA2*		*P* value for heterogeneity^a^	*I*^2^ (%)
	OR (95% CI)^b^	*P* value	OR (95% CI)	*P* value		
Biliary tract	17.4 (5.8-51.9)	2.96 × 10^−7^	NA^c^	NA	NA	NA
Breast						
Female	16.1 (7.1-36.7)	3.50 × 10^−11^	10.9 (7.0-17.1)	1.98 × 10^−25^	.41	0.0
Male	NA	NA	67.9 (19.2-239.8)	5.74 × 10^−11^	NA	NA
Cervical	NA	NA	3.2 (1.3-7.8)	8.79 × 10^−3^	NA	NA
Colorectal	1.9 (0.8-4.5)	.14	1.0 (0.5-1.9)	.99	.24	28.9
Endometrial	NA	NA	4.0 (1.6-9.9)	2.34 × 10^−3^	NA	NA
Esophageal	NA	NA	5.6 (2.9-11.0)	4.83 × 10^−7^	NA	NA
Gastric	5.2 (2.6-10.5)	3.40 × 10^−6^	4.7 (3.1-7.1)	1.37 × 10^−13^	.79	0
Liver	NA	NA	2.4 (1.1-5.0)	.02	NA	NA
Lung	3.7 (1.6-8.8)	3.08 × 10^−3^	1.7 (0.9-3.3)	.13	.16	48.6
Lymphoma	7.7 (2.6-22.4)	1.96 × 10^−4^	1.2 (0.3-5.0)	.79	.04	76.0
Ovarian	75.6 (31.6-180.6)	2.26 × 10^−22^	11.3 (5.6-23.0)	1.74 × 10^−11^	9.00 × 10^−4^	90.9
Pancreatic	12.6 (3.7-42.8)	4.67 × 10^−5^	10.7 (5.1-22.6)	4.69 × 10^−10^	.82	0
Prostate	1.1 (0.3-3.4)	.92	4.0 (2.5-6.5)	7.22 × 10^−9^	.04	77.4
Kidney	NA	NA	4.5 (1.4-14.2)	.01	NA	NA

Abbreviations: NA, not applicable, OR, odds ratio.

^a^ The heterogeneity of the association between *BRCA1* and *BRCA2* was evaluated by the Cochran Q statistic and *I^2^* statistic in the random-effects model.

^b^ We calculated cancer risk using selected patients with cancer without a family history to minimize overestimation of ORs. A logistic regression analysis using a dominant model was used with age at diagnosis for cases and age at registration for controls included as covariates.

^c^ When the number of patients in each category was fewer than 3, this calculation was not done.

### Lifetime Cumulative Risk of Each Cancer Type

**Figure 2.**
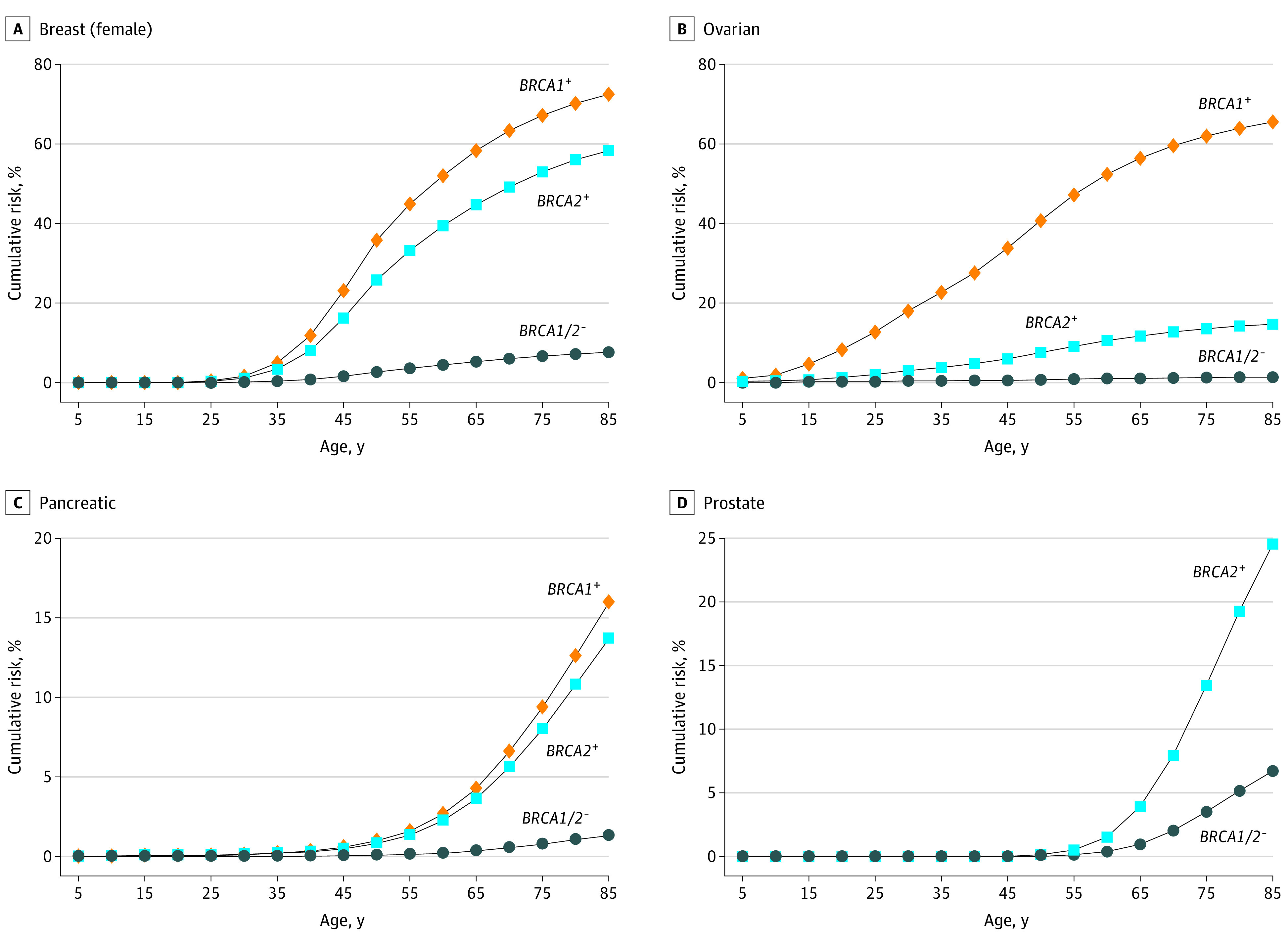
Estimated Absolute Risk of the 4 Known Associated Cancer Types The cumulative risks of each cancer to age 85 years were estimated for carriers and noncarriers of pathogenic variants in *BRCA1* and *BRCA2*. Only cancer type gene associations with *P* < 1 × 10^−4^ were described. *BRCA1*^+^ indicates *BRCA1* pathogenic variant carriers; *BRCA2*^+^, *BRCA2* pathogenic variant carriers; *BRCA1/2*^−^, individuals without a *BRCA1* and *BRCA2* pathogenic variant.

**Figure 3.**
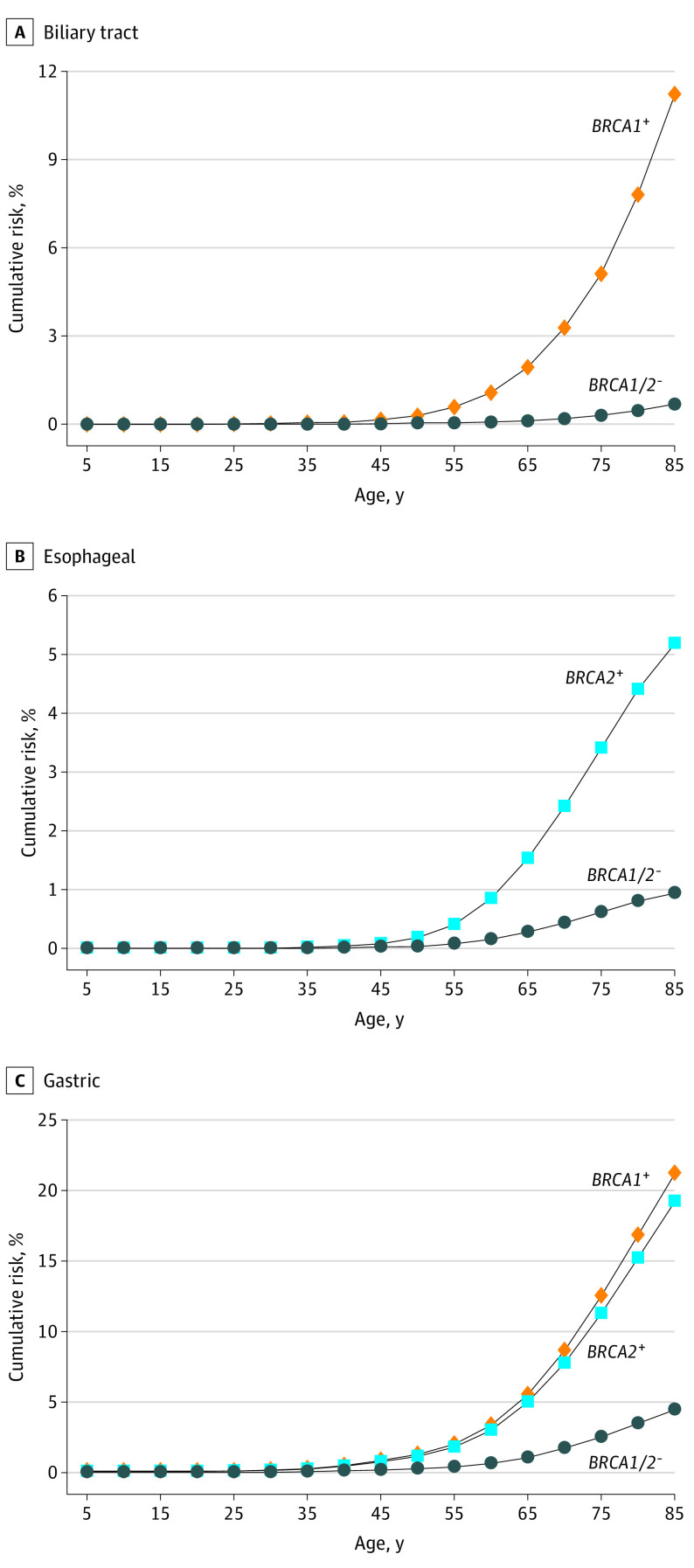
Estimated Absolute Risk of the 3 Newly Associated Cancer Types The cumulative risks of each cancer to age 85 years were estimated for carriers and noncarriers of pathogenic variants in *BRCA1* and *BRCA2*. Only cancer type gene associations with *P* < 1 × 10^−4^ were described. *BRCA1*^+^ indicates *BRCA1* pathogenic variant carriers; *BRCA2*^+^, *BRCA2* pathogenic variant carriers;* BRCA1/2*^−^, individuals without a *BRCA1* and *BRCA2* pathogenic variant.

The cumulative risk of cancer to age 85 years was estimated for carriers and noncarriers of pathogenic variants in *BRCA1* and/or *BRCA2* for the 7 significantly associated cancer types (Figures 2 and 3). In *BRCA1*, breast cancer showed the highest cumulative risk at 72.5% (95% CI, 20.4%-90.5%) followed by ovarian cancer at 65.6% (95% CI, 12.8%-86.4%), gastric cancer at 21.3% (95% CI, 6.9%-33.4%), pancreatic cancer at 16.0% (95% CI, −3.9% to 32.1%), and biliary tract cancer at 11.2% (95% CI, −1.1% to 22.1%). In *BRCA2*, the highest cumulative risk was also breast cancer at 58.3% (95% CI, 38.3%-71.9%), followed by prostate cancer at 24.5% (95% CI, 6.9%-38.8%), gastric cancer at 19.3% (95% CI, 11.9%-26.0%), ovarian cancer at 14.8% (95% CI, 4.6%-23.9%), pancreatic cancer at 13.7% (95% CI, 3.7%-22.8%), and esophageal cancer at 5.2% (95% CI, 1.7%-8.5%).

### Demographic and Clinical Characteristics of Carriers in the 7 Cancer Types

We investigated the association between carrier status and age at diagnosis for the 7 associated cancers. eTable 4 in the Supplement suggests that pathogenic variants in *BRCA1* were associated with earlier age at diagnosis of female breast cancer (−5.7 years). Meanwhile, those with *BRCA2* were associated with earlier age at diagnosis of female breast cancer (−5.7 years) and prostate cancer (−2.2 years). Carriers with *BRCA2* pathogenic variants showed a later diagnosis of ovarian cancer (4.1 years). The proportion of pathogenic variants according to diagnosis age (by 10-year age group) is shown in eFigure 6 in the Supplement.

eFigure 7 in the Supplement describes associations between the carrier status in *BRCA1 *and *BRCA2* for cases with a diagnosis of the 7 associated cancers and reported family history of each of these cancer types. For *BRCA1*, family history of ovarian cancer was strongly enriched in female breast, ovarian, and pancreatic cancers. However, for *BRCA2*, family history of breast cancer was broadly enriched in 5 cancer types.

We also investigated whether carrier patients had specific histological subtypes. We observed a different distribution of histological subtypes only for breast and ovarian cancer, which is largely consistent with previous reports^33^ (eTable 5 in the Supplement).

### Reported Family History According to Pathogenic Variant Status

We investigated the extent to which carriers with pathogenic variants were enriched for reported family history (none, 1, or 2 or more of these 7 cancer types) with the Cochran-Armitage test. Biliary tract, female breast, ovarian, and prostate cancers showed increasing enrichment of carrier patients according to the increased number of reported cancer types in relatives (eFigure 8 in the Supplement).

## Discussion

This large-scale registry-based case-control study analyzed *BRCA1 *and *BRCA2* in 63 828 patients with 14 cancer types and 37 086 controls. The proportion of pathogenic variants varied across different regions in Japan, mainly because of differences in the proportion of founder pathogenic variants. We observed that biliary tract, esophageal, and gastric cancer were significantly associated with *BRCA1* and/or *BRCA2* pathogenic variant status in addition to the 4 established cancer types. Six other cancer types showed an association. Patients with pathogenic variants were more likely to report a family history of the 7 associated cancer types.

The results of this large-scale registry-based study suggest that pathogenic variants in *BRCA1* and/or *BRCA2* are associated with increased risk of biliary tract, gastric, and esophageal cancers. Further studies are needed to reveal the mechanisms linking pathogenic variants and these cancer types for the potential efficacy of PARP inhibitors because homologous recombination repair defects were observed in some patients with biliary and esophageal cancers.^34^ These cancers are known to have a higher incidence rate in East Asian countries.^35^ The estimated lifetime cumulative risks of breast and ovarian cancer in *BRCA1 *and *BRCA2* are broadly consistent with the previous estimation^36^; the present study could not detect that the cumulative risks of ovarian cancer were low up to age 40 years for *BRCA1* carriers and age 50 years for *BRCA2* carriers because we could not calculate age-specific estimated ORs. Cumulative risk of prostate cancer for *BRCA2* carriers was lower than that estimated in the UK and Ireland,^37^ probably because prostate cancer incidence rate is higher in European countries.^35^ Conversely, cumulative risk of gastric cancer was estimated at around 20% for both genes, which is likely higher than for European populations because of the higher incidence rate in East Asia countries.^35^ Taken together, the cumulative risk for each cancer type would be associated with the different incidence rate in each country. We also observed associations with risk of 6 additional cancer types. The results suggest that the range of cancer types associated with pathogenic variants in *BRCA1 *and *BRCA2* is likely broader than that determined from previous analysis of largely European ancestry cohorts.

We observed a large difference in the carrier frequency between the 7 regions. This could be largely explained by the distribution of founder pathogenic variants. Founder pathogenic variants are known to be associated with carrier frequency in a population, which could change the best strategy for genetic tests^38^; regional differences should also be considered when designing a suitable strategy among genetic tests for selected patients, all patients, or all unaffected individuals. It also suggests that population-matched and region-matched controls would be indispensable for precise risk estimation of cancer predisposition genes rather than ExAC and gnomAD.^39^ Taken together, more detailed information that accounts for populations and regions would improve precision medicine with genetic testing of *BRCA1 *and *BRCA2*.

These risk association findings, together with our analysis of an association with family history of cancer and clinical phenotypes, are relevant for developing and adapting guidelines about genetic testing, treatment options, and treatability with PARP inhibitors for each cancer type. Depending on the cancer type, different guidelines exist to prioritize patients for receiving germline gene testing. The National Comprehensive Cancer Network guidelines^38^ recommend that all patients with pancreatic cancer should be tested. Meanwhile, patients with breast or ovarian cancer are selected based on several criteria. This study suggests that the family history of the 7 associated cancer types efficiently identified patients with pathogenic variants. Therefore, this information would be useful to expand indications for genetic testing of individuals with family history of these cancer types.

### Limitations

This study has several limitations. We selected controls without family history of cancer because we intended to improve the statistical power for association analysis and limit the effect of family history, including shared genetic and environmental effects.^40^ This would affect the generalizability of the study results. However, the estimated cumulative risks were comparable with those based on prospective cohorts, suggesting that the study design did not greatly affect the results. We analyzed only single-nucleotide variants and small indels, but structural variants are known to be associated with hereditary breast cancer.^41^ However, the proportion of pathogenic variants due to structural changes is reported to be very low for the *BRCA1 *and *BRCA2* genes in Japanese populations.^42^ Lastly, we tested for a linear association in several statistical analyses; however, we had the potential to miss other patterns of association.^43^ In particular, biliary tract cancer in *BRCA2* (eFigure 6 in the Supplement) showed an unusual pattern that should be investigated in further studies.

## Conclusions

This large-scale registry-based case-control study of 63 828 patients across 14 cancer types and 37 086 population-matched and region-matched controls provided a broad view of carrier frequency, disease risk, family history, and clinical characteristics of pathogenic variant carriers. This information can potentially improve genetic testing of *BRCA1 *and *BRCA2* for various cancer types for Asian countries and encourage similar research in other countries.

## Notes

Supplement. eAppendix. eFigure 1. Overall procedure for this study eFigure 2. Location and the number of pathogenic variants in BRCA1 and BRCA2 in patients eFigure 3. Distribution of carrier frequency in patients between seven regions of Japan in (A) BRCA1 and (B) BRCA2 eFigure 4. Carrier frequency in patients with multiple cancer diagnoses, for the 14 cancer types eFigure 5. Comparison of the carrier frequency of the 14 cancer types and controls between females and males in (A) BRCA1 and (B) BRCA2 eFigure 6. The proportion of patients with pathogenic variants by the age at diagnosis of each cancer type in 10-year age groupings eFigure 7. Associations between carrier status of pathogenic variants in BRCA1 or BRCA2 and family history eFigure 8. The combined BRCA1 or BRCA2 carrier frequency of patients according to family history of the seven cancer types eTable 1. List of the 315 pathogenic variants in BRCA1 and BRCA2 eTable 2. Comparison of BRCA1 and BRCA2 pathogenic variant frequency for all* patients for each cancer type, versus controls eTable 3. Results of two sensitivity analyses in breast cancer eTable 4. Mean age at diagnosis of each cancer type in patients with or without pathogenic variants in BRCA1 and BRCA2 eTable 5. Comparisons of histological subtypes between carriers with pathogenic variants and non-carriers
