# Supplementary material for: Expansion of Cancer Risk Profile for BRCA1 and BRCA2 Pathogenic Variants
Source: JAMA Oncol. 2022 Apr 14;8(6):871–8. doi: 10.1001/jamaoncol.2022.0476 (PMC9011177; doi:10.1001/jamaoncol.2022.0476)
Supplement: Supplement. — eAppendix. eFigure 1. Overall procedure for this study eFigure 2. Location and the number of pathogenic variants in BRCA1 and BRCA2 in patients eFigure 3. Distribution of carrier frequency in patients between seven regions of Japan in (A) BRCA1 and (B) BRCA2 eFigure 4. Carrier frequency in patients with multiple cancer diagnoses, for the 14 cancer types eFigure 5. Comparison of the carrier frequency of the 14 cancer types and controls between females and males in (A) BRCA1 and (B) BRCA2 eFigure 6. The proportion of patients with pathogenic variants by the age at diagnosis of each cancer type in 10-year age groupings eFigure 7. Associations between carrier status of pathogenic variants in BRCA1 or BRCA2 and family history eFigure 8. The combined BRCA1 or BRCA2 carrier frequency of patients according to family history of the seven cancer types eTable 1. List of the 315 pathogenic variants in BRCA1 and BRCA2 eTable 2. Comparison of BRCA1 and BRCA2 pathogenic variant frequency for all* patients for each cancer type, versus controls eTable 3. Results of two sensitivity analyses in breast cancer eTable 4. Mean age at diagnosis of each cancer type in patients with or without pathogenic variants in BRCA1 and BRCA2 eTable 5. Comparisons of histological subtypes between carriers with pathogenic variants and non-carriers [file jamaoncol-e220476-s001.pdf]

Supplemental Online Content

Momozawa Y, Sasai R, Usui Y, et al. Expansion of cancer risk profile for *BRCA1* and *BRCA2* pathogenic variants. *JAMA Oncology*. Published online April 14, 2022. doi:10.1001/jamaoncol.2022.0476

eAppendix.
eFigure 1. Overall procedure for this study
eFigure 2. Location and the number of pathogenic variants in BRCA1 and BRCA2 in patients
eFigure 3. Distribution of carrier frequency in patients between seven regions of Japan in (A) BRCA1 and (B) BRCA2
eFigure 4. Carrier frequency in patients with multiple cancer diagnoses, for the 14 cancer types
eFigure 5. Comparison of the carrier frequency of the 14 cancer types and controls between females and males in (A) BRCA1 and (B) BRCA2
eFigure 6. The proportion of patients with pathogenic variants by the age at diagnosis of each cancer type in 10-year age groupings
eFigure 7. Associations between carrier status of pathogenic variants in BRCA1 or BRCA2 and family history
eFigure 8. The combined BRCA1 or BRCA2 carrier frequency of patients according to family history of the seven cancer types
eTable 1. List of the 315 pathogenic variants in BRCA1 and BRCA2
eTable 2. Comparison of BRCA1 and BRCA2 pathogenic variant frequency for all* patients for each cancer type, versus controls
eTable 3. Results of two sensitivity analyses in breast cancer
eTable 4. Mean age at diagnosis of each cancer type in patients with or without pathogenic variants in BRCA1 and BRCA2
eTable 5. Comparisons of histological subtypes between carriers with pathogenic variants and non-carriers

This supplemental material has been provided by the authors to give readers additional information about their work.

© 2022 Momozawa Y et al. *JAMA Oncology.*

eAppendix.

1. Assignment of clinical significance for all variants

We assigned clinical significance (pathogenic, likely pathogenic, benign, likely benign or uncertain) for all variants using *BRCA1/2* variant classification criteria (https://variansci.files.wordpress.com/2018/10/enigma-brca12-gene-variant-classification-criteria_v2-5-1.pdf) previously developed by members of the ENIGMA (Evidence-based Network for the Interpretation of Germline Mutant Alleles) consortium[1], in their role as a ClinGen-approved external expert panel for *BRCA1/2*. Pathogenic and likely pathogenic variants were collectively referred to as pathogenic variants in this study.

2. Calculation for the lifetime cumulative risk of each cancer type

We estimated the cumulative risk for each cancer by using the odds ratio of each category, the prevalence of each category among our study control, the age-specific cancer incidence rate of Japan[2], and the age-specific population rate of Japan in 2017[2]. We assumed that the site-specific prevalence of each category was the same in each age group since the presence or absence of the pathogenic variants did not change after birth, and also assumed that the distribution of the pathogenic variants for each cancer in the Japanese general population was represented by that in our study control sample[3-5].

© 2022 Momozawa Y et al. *JAMA Oncology.*

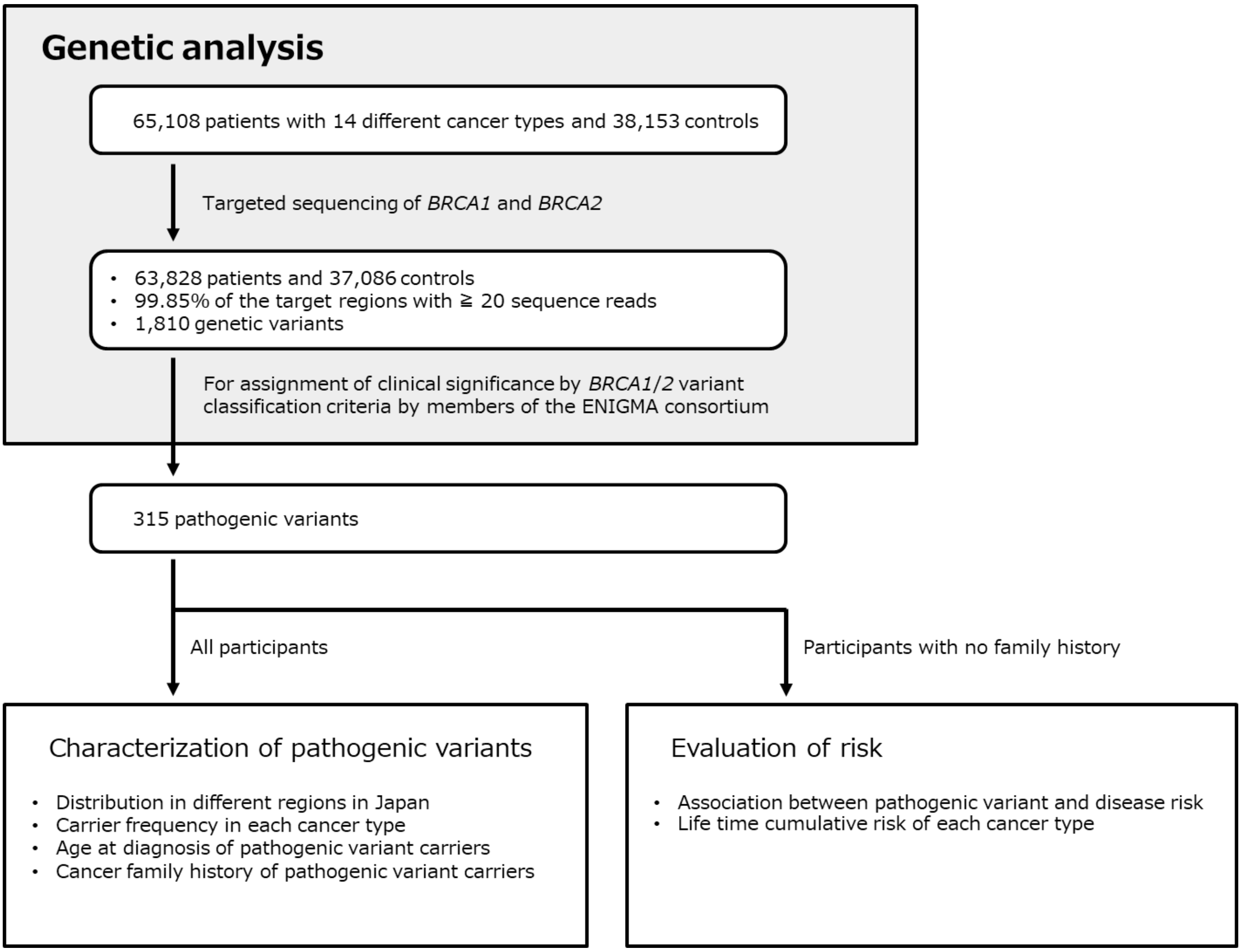

eFigure 1. Overall procedure for this study

© 2022 Momozawa Y et al. *JAMA Oncology.*

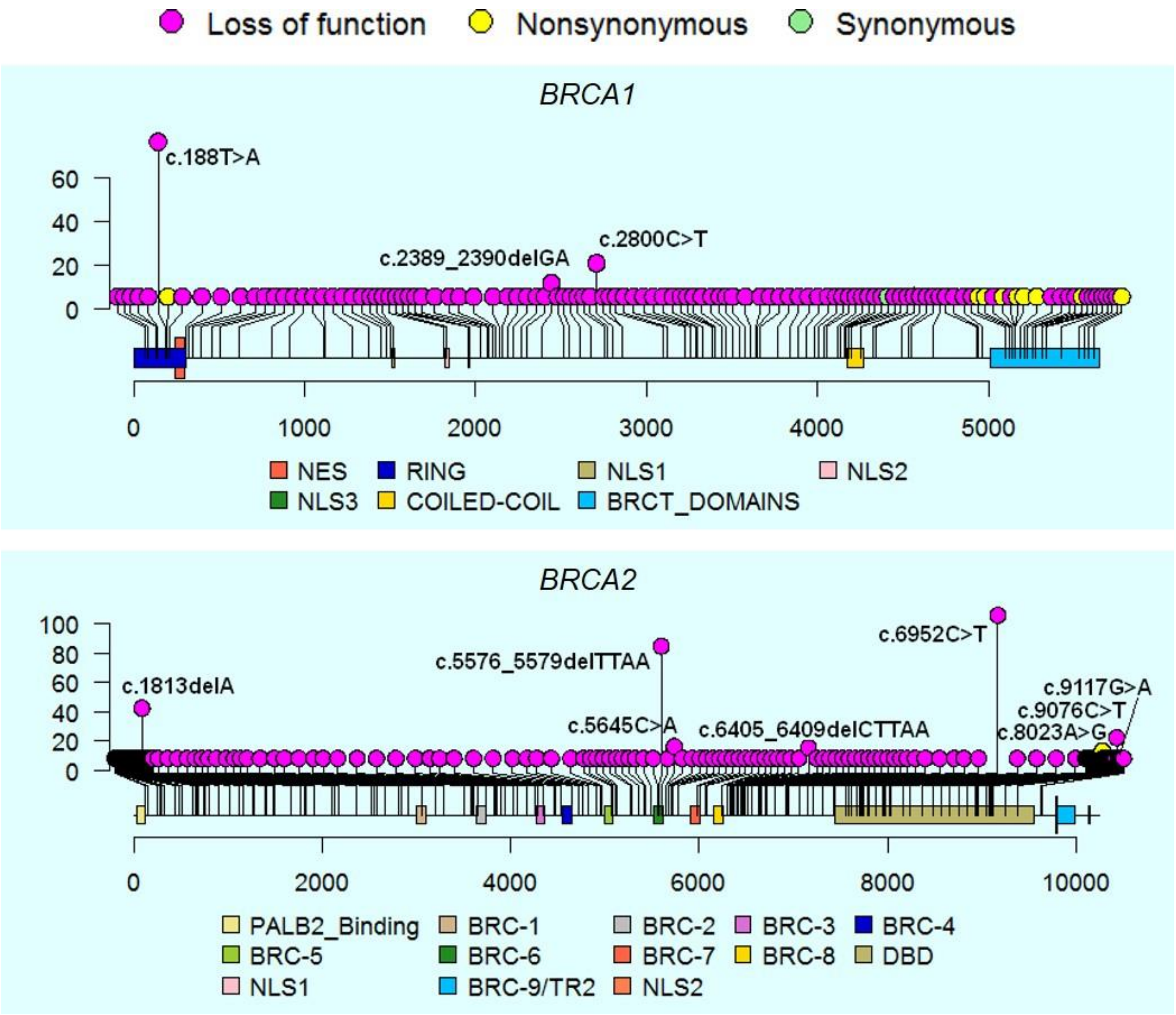

eFigure 2. Location and the number of pathogenic variants in *BRCA1* and *BRCA2* in patients.

Locations of pathogenic variants found in patients and protein domains are shown by lollipop structures with the variant type indicated by color. Protein domains designated in the ENIGMA *BRCA1/2* gene variant classification criteria (eAppendix 1) are described. HGVS.c of frequent variants with ≥ 10 patients is shown. Three pathogenic variants were shared in ≥ 10 patients in *BRCA1* (c.188T>A in 78 patients, c.2389_2390delGA in 14 patients, and c.2800C>T in 23 patients), and eight pathogenic variants for *BRCA2* (c.1813delA in 45 patients, c.5576_5579delTTAA in 87 patients, c.5645C>A in 19 patients, c.6405_6409delCTTAA in 18 patients, c.6952C>T in 108 patients, c.8023A>G in 16 patients, c.9076C>T in 25 patients, and c.9117G>A in 10 patients).

© 2022 Momozawa Y et al. *JAMA Oncology.*

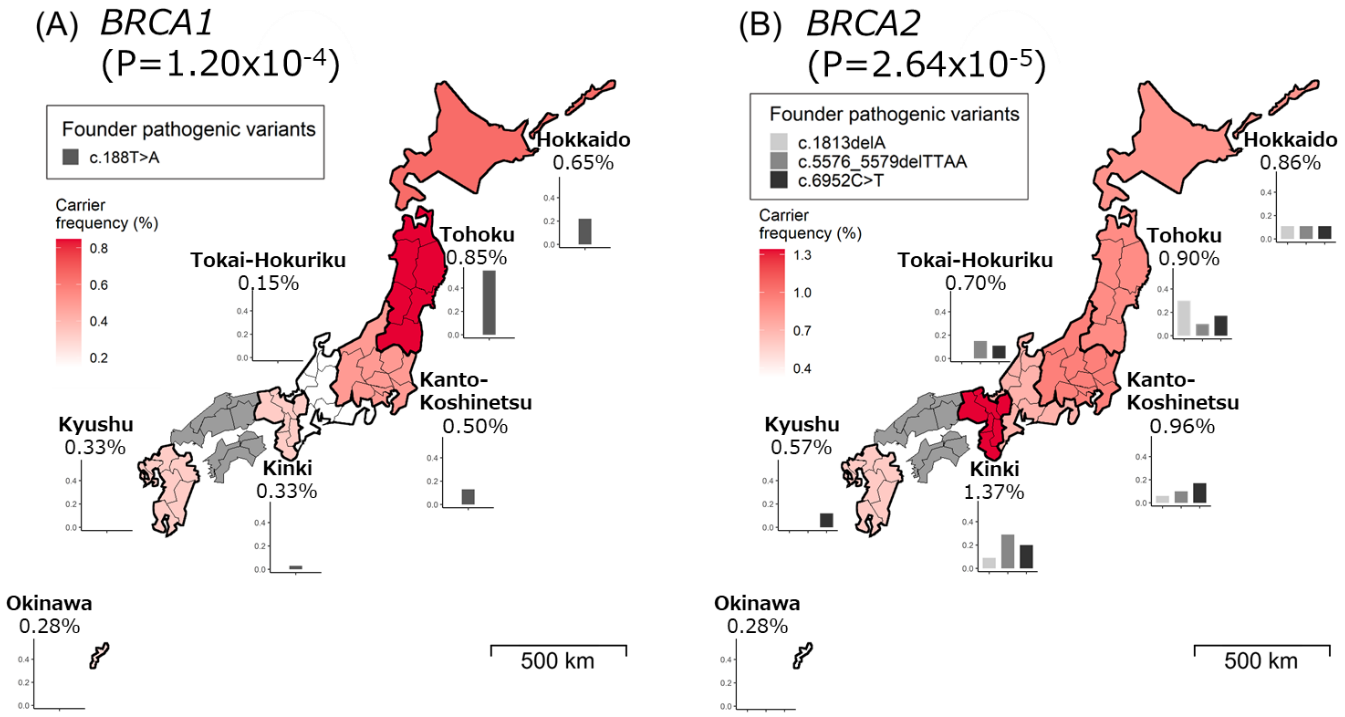

eFigure 3. Distribution of carrier frequency in patients between seven regions of Japan in (A) *BRCA1* and (B) *BRCA2*.

The carrier frequency is shown as a heat map between white and red. P value was calculated by χ^2 test. Grey shows the region in which we did not analyze the samples. In each region, the region name, frequency of pathogenic variants, the histogram for the frequency of one (c.188T>A) in *BRCA1* or three founder pathogenic variants (c.1813delA, c.5576_5579delTTAA, and c.6952C>T) in *BRCA2* are shown. Regional differences could be largely explained by the different proportion of founder pathogenic variants (shown as histograms). *BRCA1* c.188T>A was observed in 0.55% of patients from Tohoku, but was absent from patients from Tokai-Hokuriku. The total proportion of the three top founder *BRCA2* pathogenic variants (c.1813delA, c.5576_5579delTTAA, and c.6952C>T) ranged from 0.58% in Kinki to 0% in Okinawa.

© 2022 Momozawa Y et al. *JAMA Oncology.*

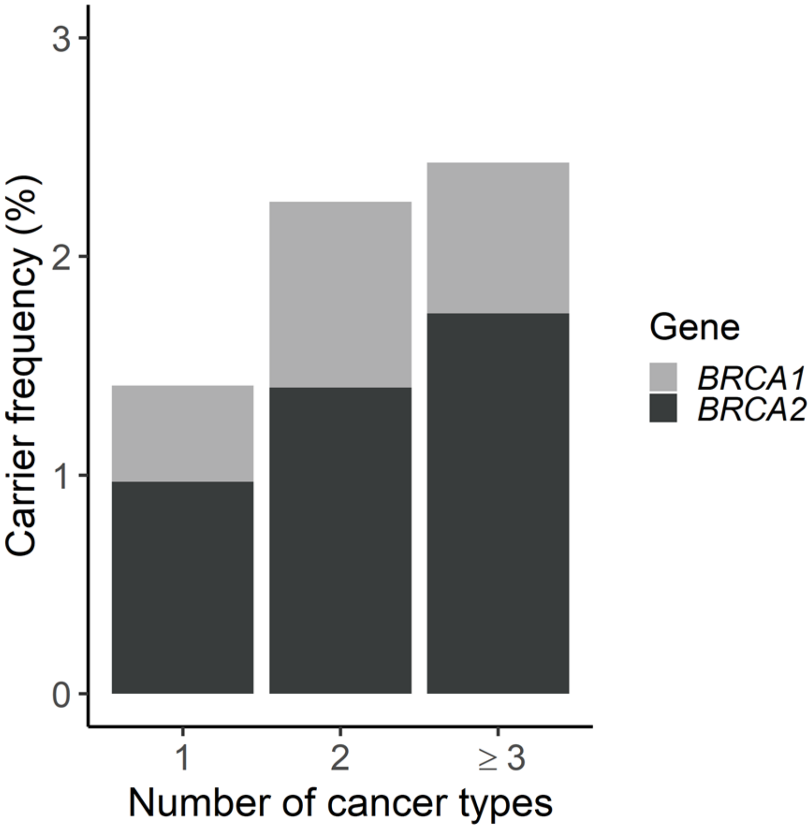

eFigure 4. Carrier frequency in patients with multiple cancer diagnoses, for the 14 cancer types.

For both genes, carrier frequency significantly increased according to the number of cancer types diagnosed for a patient (P = 7.73×10^{-4} in *BRCA1* and P = 4.90×10^{-3} in *BRCA2* by the Cochran-Armitage test).

© 2022 Momozawa Y et al. *JAMA Oncology.*

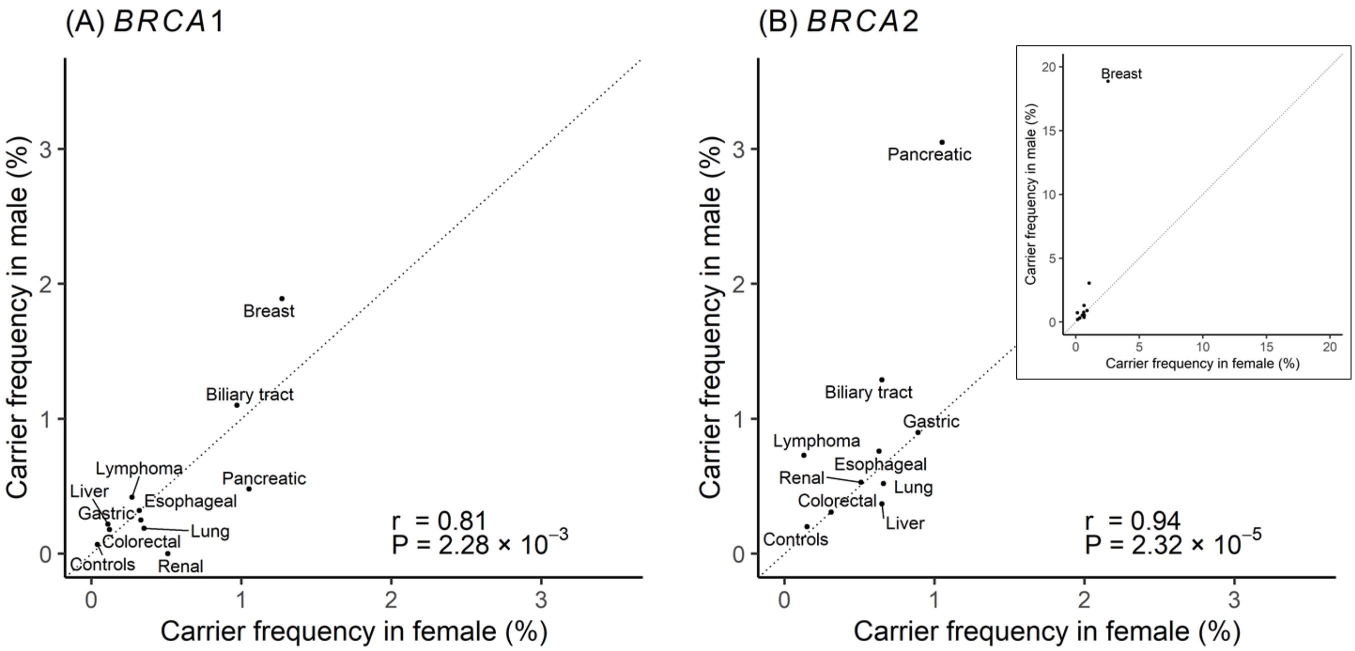

eFigure 5. Comparison of the carrier frequency of the 14 cancer types and controls between females and males in (A) *BRCA1* and (B) *BRCA2*.
Carrier frequency for females and males in each cancer type was significantly correlated. The high carrier frequency of pathogenic variants in *BRCA2* is comparable to that of other populations[6].

© 2022 Momozawa Y et al. *JAMA Oncology.*

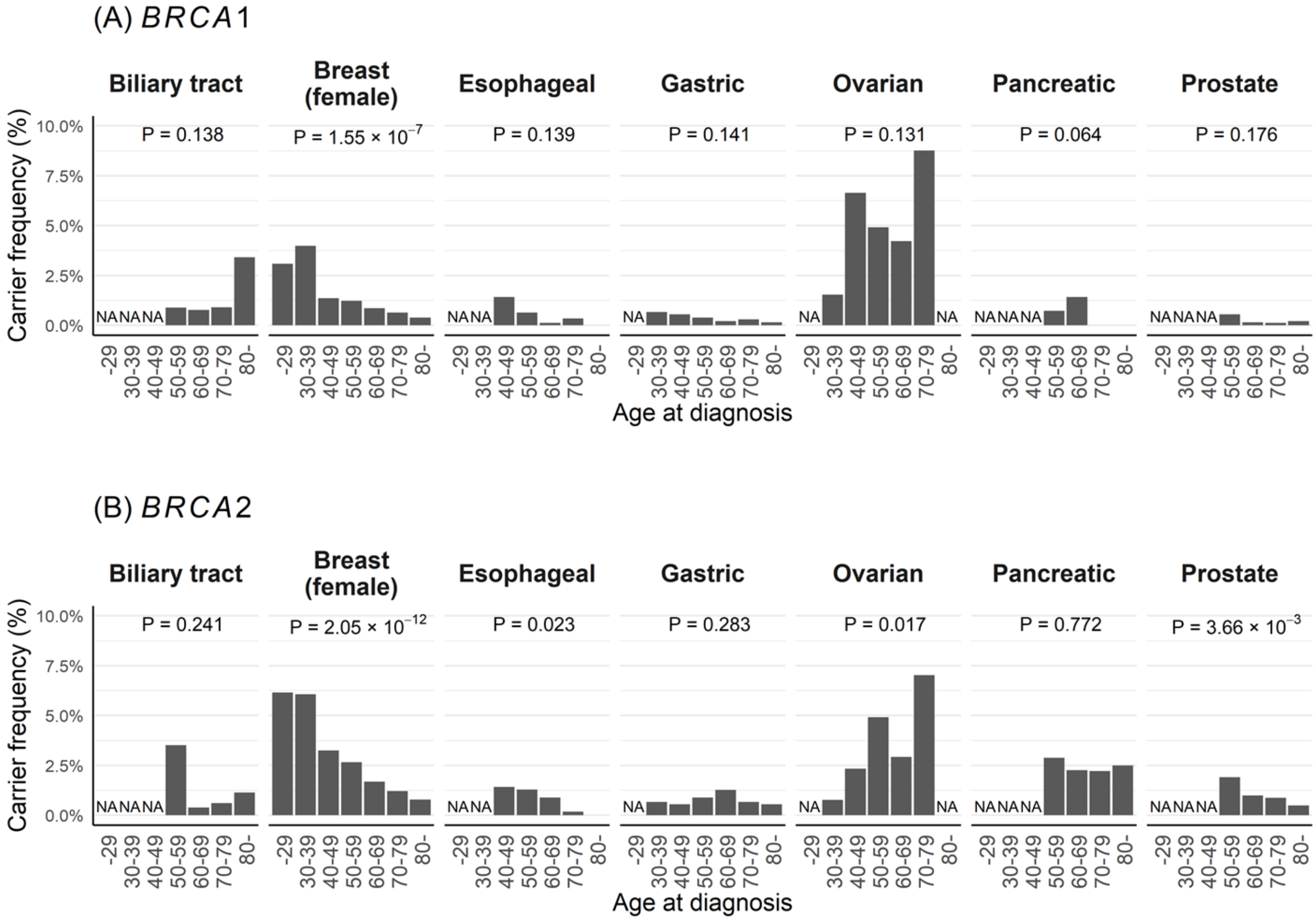

eFigure 6. The proportion of patients with pathogenic variants by the age at diagnosis of each cancer type in 10-year age groupings.
Data are shown in the 10-year age groups with 50 or more patients. The P value was calculated using the Cochran-Armitage test to test for a linear association. P = 7.14 x 10^{-3} was set at the threshold of significance.

© 2022 Momozawa Y et al. *JAMA Oncology.*

(A) *BRCA1*

Family history (columns) × Patient cancer type (rows)

Patient cancer type	Biliary tract	Breast	Esophageal	Gastric	Ovarian	Pancreatic	Prostate
Biliary tract	4.9 (0.1 - 39.3) 0.211	5.2 (0.5 - 28.2) 0.081	5.1 (0.1 - 41.5) 0.202	0.4 (0.0 - 3.4) 0.692	0.0 (0.0 - 115.3) 1.000	3.0 (0.1 - 23.2) 0.316	0.0 (0.0 - 26.8) 1.000
Breast (female)	1.0 (0.1 - 3.9) 0.722	3.9 (2.6 - 5.8) 1.05×10^{-10}	1.1 (0.3 - 3.0) 0.784	1.4 (0.9 - 2.2) 0.075	10.1 (5.2 - 18.3) 1.30×10^{-9}	1.1 (0.3 - 2.6) 0.809	0.4 (0.0 - 1.5) 0.244
Esophageal	11.0 (0.2 - 95.3) 0.102	8.4 (0.8 - 52.1) 0.038	2.6 (0.1 - 21.3) 0.358	0.6 (0.0 - 5.4) 1.000	44.2 (0.9 - 435.4) 0.029	0.0 (0.0 - 17.0) 1.000	0.0 (0.0 - 28.9) 1.000
Gastric	5.9 (0.7 - 23.8) 0.051	5.3 (1.8 - 13.5) 2.01×10^{-3}	0.0 (0.0 - 4.0) 1.000	0.8 (0.3 - 2.0) 0.836	11.1 (1.3 - 45.7) 0.016	1.0 (0.0 - 6.2) 1.000	0.0 (0.0 - 6.3) 1.000
Ovarian	3.8 (0.7 - 13.7) 0.061	2.7 (1.3 - 5.4) 4.49×10^{-3}	1.9 (0.4 - 6.4) 0.227	1.1 (0.6 - 2.0) 0.761	14.5 (7.3 - 28.2) 3.17×10^{-13}	1.4 (0.4 - 4.1) 0.528	0.0 (0.0 - 1.7) 0.166
Pancreatic	0.0 (0.0 - 38.2) 1.000	2.6 (0.1 - 21.5) 0.361	0.0 (0.0 - 19.8) 1.000	0.0 (0.0 - 2.4) 0.360	75.9 (5.9 - 649.8) 8.63×10^{-4}	0.0 (0.0 - 8.2) 1.000	0.0 (0.0 - 20.4) 1.000
Prostate	0.0 (0.0 - 19.0) 1.000	3.9 (0.7 - 13.8) 0.055	0.0 (0.0 - 8.8) 1.000	1.8 (0.5 - 5.3) 0.350	29.9 (3.2 - 133.4) 2.68×10^{-3}	1.6 (0.0 - 10.2) 0.481	1.4 (0.2 - 6.0) 0.655

(B) *BRCA2*

Family history (columns) × Patient cancer type (rows)

Patient cancer type	Biliary tract	Breast	Esophageal	Gastric	Ovarian	Pancreatic	Prostate
Biliary tract	0.0 (0.0 - 20.2) 1.000	15.1 (2.9 - 73.1) 7.71×10^{-4}	5.1 (0.1 - 41.5) 0.202	4.6 (1.0 - 23.4) 0.027	0.0 (0.0 - 115.3) 1.000	0.0 (0.0 - 12.2) 1.000	0.0 (0.0 - 26.8) 1.000
Breast (female)	1.3 (0.4 - 3.2) 0.443	3.0 (2.2 - 4.0) 7.06×10^{-12}	1.6 (0.8 - 2.9) 0.172	1.3 (1.0 - 1.8) 0.068	3.6 (1.7 - 6.7) 5.81×10^{-4}	2.8 (1.7 - 4.3) 4.87×10^{-5}	1.9 (1.1 - 3.1) 0.019
Esophageal	0.0 (0.0 - 17.5) 1.000	1.4 (0.0 - 9.2) 0.531	0.0 (0.0 - 4.0) 0.619	0.9 (0.2 - 3.3) 1.000	0.0 (0.0 - 74.1) 1.000	1.6 (0.0 - 10.8) 0.478	0.0 (0.0 - 10.8) 1.000
Gastric	0.8 (0.0 - 4.8) 1.000	3.3 (1.7 - 5.9) 4.92×10^{-4}	1.0 (0.2 - 2.9) 1.000	1.2 (0.8 - 1.9) 0.359	6.8 (1.8 - 18.7) 3.88×10^{-3}	1.2 (0.3 - 3.3) 0.567	0.5 (0.0 - 2.8) 0.725
Ovarian	0.0 (0.0 - 6.2) 1.000	3.8 (1.7 - 7.9) 6.96×10^{-4}	0.9 (0.0 - 5.3) 1.000	1.6 (0.8 - 3.1) 0.148	3.2 (0.9 - 8.4) 0.032	1.5 (0.3 - 5.0) 0.451	3.8 (1.1 - 10.3) 0.017
Pancreatic	0.0 (0.0 - 9.6) 1.000	4.5 (1.3 - 13.1) 0.011	1.3 (0.0 - 8.3) 0.562	1.9 (0.7 - 4.8) 0.203	0.0 (0.0 - 31.1) 1.000	1.8 (0.3 - 6.2) 0.418	1.3 (0.0 - 8.6) 0.552
Prostate	1.5 (0.2 - 5.7) 0.389	3.3 (1.8 - 5.7) 1.06×10^{-4}	0.7 (0.1 - 2.6) 1.000	0.8 (0.5 - 1.4) 0.536	0.0 (0.0 - 7.9) 1.000	4.3 (2.3 - 7.5) 1.01×10^{-5}	1.9 (1.0 - 3.2) 0.024

OR
(95%CI)
P value

© 2022 Momozawa Y et al. *JAMA Oncology.*

eFigure 7. Associations between carrier status of pathogenic variants in *BRCA1 or BRCA2* and family history.

Fisher's exact test was used. P = was 1.02×10^{-3} considered as the threshold of significance by the Bonferroni correction. Red indicates $P < 1.02 \times 10^{-3}$, and deep red shows OR ≥ 4 in addition to $P < 1.02 \times 10^{-3}$.

© 2022 Momozawa Y et al. *JAMA Oncology.*

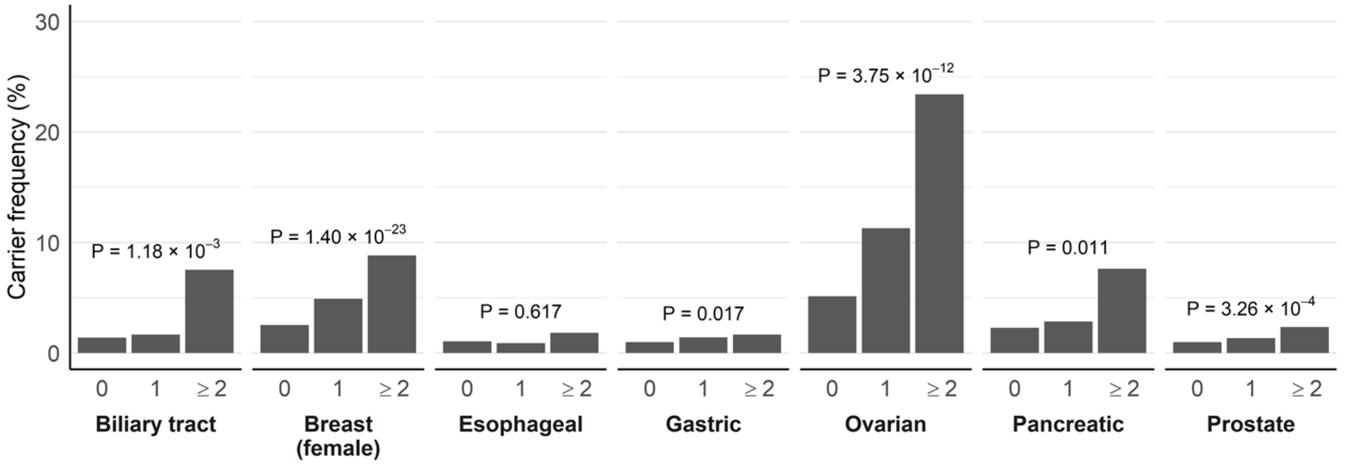

eFigure 8. The combined *BRCA1* or *BRCA2* carrier frequency of patients according to family history of the seven cancer types.

Carrier frequency with patients the seven associated cancer types was compared for individuals without family history on any of the seven cancer types versus those with reported family history of 1 cancer type, or with family history of ≥ 2 cancer types. The P value was calculated using the Cochran-Armitage test to test for a linear association. P = 7.14 x 10^{-3} was set at the threshold of significance. Calculations for male breast cancer were not done due to the low number of patients.

© 2022 Momozawa Y et al. *JAMA Oncology.*

4. eTables

eTable 1. List of the 315 pathogenic variants in *BRCA1* and *BRCA2*

Chromosome	Position	Reference allele	Alternative allele	dbSNP151	Gene	Annotation	HGVS.c	HGVS.p	Carrier frequency in patients (%)	Carrier frequency in controls (%)	Call rate (%)	P value for Hardy-Weinberg equilibrium in controls	Average depth
13	32,893,269	CTATAA	C	.	*BRCA2*	frameshift variant	c.125_129delATAAT	p.Tyr42fs	0.002	0	100	1	2,069
13	32,893,291	G	T	rs80358435	*BRCA2*	stop gained	c.145G>T	p.Glu49*	0.002	0	100	1	2,948
13	32,893,393	G	T	rs886040428	*BRCA2*	stop gained	c.247G>T	p.Glu83*	0.002	0	100	1	2,796
13	32,893,420	C	T	rs80358529	*BRCA2*	stop gained	c.274C>T	p.Gln92*	0.002	0	100	1	878
13	32,893,425	TC	T	.	*BRCA2*	frameshift variant	c.281delC	p.Pro94fs	0.002	0	100	1	878
13	32,893,435	G	T	rs397507646	*BRCA2*	stop gained	c.289G>T	p.Glu97*	0.003	0	100	1	878
13	32,893,463	G	A	rs397507303	*BRCA2*	splice donor variant & intron variant	c.316+1G>A		0.002	0	100	1	878
13	32,893,464	T	C	rs81002805	*BRCA2*	splice donor variant & intron variant	c.316+2T>C		0.005	0.003	100	1	878
13	32,900,251	C	T	rs397507717	*BRCA2*	stop gained	c.439C>T	p.Gln147*	0.002	0	100	1	1,221

© 2022 Momozawa Y et al. *JAMA Oncology.*

13	32,900,288	G	A	rs81002797	*BRCA2*	splice donor variant & intron variant	c.475+1G>A		0.013	0	100	1	1,222
13	32,900,386	T	A	.	*BRCA2*	stop gained	c.483T>A	p.Cys161*	0.002	0	100	1	1,980
13	32,900,421	T	C	.	*BRCA2*	splice donor variant & intron variant	c.516+2T>C		0.003	0	100	1	990
13	32,903,579	G	A	rs81002820	*BRCA2*	Splice acceptor variant & intron variant	c.632-1G>A		0.003	0	100	1	1,206
13	32,903,604	CTG	C	rs1131692273; rs80359604	*BRCA2*	frameshift variant	c.658_659delGT	p.Val220fs	0.006	0	100	1	1,206
13	32,903,612	CCT	C	.	*BRCA2*	frameshift variant	c.666_667delTC	p.His223fs	0.002	0	100	1	1,206
13	32,905,055	G	T	.	*BRCA2*	splice acceptor variant & intron variant	c.682-1G>T		0.002	0	100	1	2,356
13	32,905,109	AT	A	.	*BRCA2*	frameshift variant	c.738delT	p.Phe246fs	0.002	0	100	1	2,357
13	32,905,123	TGACA	T	rs1455701926; rs80359659	*BRCA2*	frameshift variant	c.755_758delACAG	p.Asp252fs	0.009	0	100	1	2,357
13	32,905,169	T	G	rs886040942	*BRCA2*	splice donor variant & intron variant	c.793+2T>G		0.002	0	100	1	1,177
13	32,906,410	AT	A	rs886040739	*BRCA2*	frameshift variant	c.798delT	p.Phe266fs	0.002	0	99.313	1	102
13	32,906,415	G	GA	rs886040744	*BRCA2*	frameshift variant	c.805dupA	p.Thr269fs	0.008	0	99.313	1	102
13	32,906,506	A	AAC	.	*BRCA2*	frameshift variant	c.893_894dupCA	p.Val299fs	0.002	0	99.98	1	169
13	32,906,627	GC	G	.	*BRCA2*	frameshift variant	c.1013delC	p.Ala338fs	0.003	0	99.997	1	477
13	32,906,737	GC	G	.	*BRCA2*	frameshift variant	c.1125delC	p.Phe376fs	0.002	0	100	1	672

© 2022 Momozawa Y et al. *JAMA Oncology.*

13	32,906,769	AG	A	rs397507262	*BRCA2*	frameshift variant	c.1156delG	p.Glu386fs	0	0.003	100	1	825
13	32,906,773	AG	A	.	*BRCA2*	frameshift variant	c.1159delG	p.Val387fs	0.002	0	100	1	825
13	32,906,799	G	A	rs886040347	*BRCA2*	stop gained	c.1184G>A	p.Trp395*	0	0.003	100	1	825
13	32,906,876	C	T	rs80358419	*BRCA2*	stop gained	c.1261C>T	p.Gln421*	0.002	0.003	100	1	831
13	32,906,888	GA	G	rs80359274	*BRCA2*	frameshift variant	c.1278delA	p.Asp427fs	0.014	0	100	1	831
13	32,907,003	CAG	C	rs80359283	*BRCA2*	frameshift variant	c.1389_1390delAG	p.Val464fs	0.003	0	100	1	440
13	32,907,014	A	T	rs80358427	*BRCA2*	stop gained	c.1399A>T	p.Lys467*	0.006	0.003	100	1	440
13	32,907,026	G	T	rs80358428	*BRCA2*	stop gained	c.1411G>T	p.Glu471*	0.002	0	100	1	440
13	32,907,095	GT	G	.	*BRCA2*	frameshift variant	c.1481delT	p.Val494fs	0.002	0	100	1	1,339
13	32,907,105	C	A	.	*BRCA2*	stop gained	c.1490C>A	p.Ser497*	0.005	0	100	1	1,339
13	32,907,280	AAATTT	A	.	*BRCA2*	frameshift variant	c.1670_1674delTAATT	p.Leu557fs	0.002	0	99.999	1	354
13	32,907,375	CAAAT	C	rs80359303	*BRCA2*	frameshift variant	c.1763_1766delATAA	p.Asn588fs	0.002	0	100	1	573
13	32,907,382	GT	G	.	*BRCA2*	frameshift variant	c.1770delT	p.Phe590fs	0.002	0	100	1	573
13	32,907,420	GA	G	rs80359307	*BRCA2*	frameshift variant	c.1813delA	p.Ile605fs	0.071	0.008	100	1	573
13	32,907,420	G	GA	rs1253401667; rs80359306	*BRCA2*	frameshift variant	c.1813dupA	p.Ile605fs	0.005	0	100	1	573
13	32,907,440	C	T	rs80358472	*BRCA2*	stop gained	c.1825C>T	p.Gln609*	0.002	0	100	1	513
13	32,907,525	G	A	rs587781629	*BRCA2*	splice donor variant & intron variant	c.1909+1G>A		0.003	0	100	1	412
13	32,910,588	AG	A	.	*BRCA2*	frameshift variant	c.2097delG	p.Gln699fs	0.002	0	100	1	597

© 2022 Momozawa Y et al. *JAMA Oncology.*

13	32,910,625	C	A	.	*BRCA2*	stop gained	c.2133C>A	p.Cys711*	0.002	0	100	1	241
13	32,910,672	C	CA	.	*BRCA2*	frameshift variant	c.2181dupA	p.Asp728fs	0.002	0	100	1	480
13	32,910,859	AG	A	.	*BRCA2*	frameshift variant	c.2368delG	p.Glu790fs	0.002	0	100	1	947
13	32,911,005	AATAC	A	.	*BRCA2*	frameshift variant	c.2516_2519delACAT	p.Tyr839fs	0.003	0	100	1	300
13	32,911,039	A	ACC	.	*BRCA2*	frameshift variant	c.2548_2549insCC	p.Gln850fs	0.002	0	100	1	300
13	32,911,073	C	T	.	*BRCA2*	stop gained	c.2581C>T	p.Gln861*	0.002	0	100	1	510
13	32,911,158	AT	A	.	*BRCA2*	frameshift variant	c.2670delT	p.Phe890fs	0.003	0	99.999	1	362
13	32,911,293	GT	G	.	*BRCA2*	frameshift variant	c.2802delT	p.Asp935fs	0	0.003	100	1	1,545
13	32,911,297	TAAAC	T	rs80359351	*BRCA2*	frameshift variant	c.2808_2811delACAA	p.Ala938fs	0.005	0	100	1	1,545
13	32,911,321	TA	T	rs397509342	*BRCA2*	frameshift variant	c.2835delA	p.Asp946fs	0.002	0	100	1	1,179
13	32,911,404	T	A	.	*BRCA2*	stop gained	c.2912T>A	p.Leu971*	0.002	0	100	1	1,183
13	32,911,555	AC	A	.	*BRCA2*	frameshift variant	c.3064delC	p.His1022fs	0.002	0	99.999	1	354
13	32,911,683	CAATT	C	rs80359375	*BRCA2*	frameshift variant	c.3195_3198delTAAT	p.Asn1066fs	0.002	0	100	1	230
13	32,911,693	TG	T	rs397507658	*BRCA2*	frameshift variant	c.3202delG	p.Val1068fs	0.002	0	100	1	230
13	32,911,724	GT	G	rs397507660	*BRCA2*	frameshift variant	c.3235delT	p.Ser1079fs	0	0.003	100	1	454
13	32,911,788	C	G	rs397507663	*BRCA2*	stop gained	c.3296C>G	p.Ser1099*	0.002	0	100	1	933
13	32,911,969	CAG	C	.	*BRCA2*	frameshift variant	c.3481_3482delGA	p.Asp1161fs	0	0.003	100	1	709
13	32,912,000	G	GC	.	*BRCA2*	frameshift variant	c.3512dupC	p.Ser1172fs	0.002	0	100	1	1,328
13	32,912,062	GA	G	.	*BRCA2*	frameshift variant	c.3572delA	p.Lys1191fs	0.003	0	100	1	1,235

© 2022 Momozawa Y et al. *JAMA Oncology.*

13	32,912,089	CTG	C	rs80359391	*BRCA2*	frameshift variant	c.3599_3600delGT	p.Cys1200fs	0.005	0.008	100	1	1,237
13	32,912,091	G	GTT	.	*BRCA2*	frameshift variant	c.3600_3601insTT	p.Asn1201fs	0.002	0	100	1	1,236
13	32,912,095	CA	C	.	*BRCA2*	frameshift variant	c.3607delA	p.Ser1203fs	0.002	0	100	1	1,236
13	32,912,140	TA	T	rs864622134	*BRCA2*	frameshift variant	c.3649delA	p.Arg1217fs	0.006	0	100	1	616
13	32,912,233	TAGTG	T	rs1338928084; rs80359403	*BRCA2*	frameshift variant	c.3744_3747delTGAG	p.Ser1248fs	0.002	0	100	1	751
13	32,912,318	GA	G	rs397507689	*BRCA2*	frameshift variant	c.3830delA	p.Asn1277fs	0.002	0	100	1	804
13	32,912,328	AT	A	rs80359404	*BRCA2*	frameshift variant	c.3837delT	p.Asn1279fs	0.002	0	100	1	804
13	32,912,337	CTG	C	rs1229267914; rs80359405	*BRCA2*	frameshift variant	c.3847_3848delGT	p.Val1283fs	0.002	0	100	1	855
13	32,912,387	G	T	.	*BRCA2*	stop gained	c.3895G>T	p.Glu1299*	0.002	0	100	1	855
13	32,912,432	A	T	.	*BRCA2*	stop gained	c.3940A>T	p.Lys1314*	0.005	0	99.837	1	165
13	32,912,511	AT	A	rs397507702	*BRCA2*	frameshift variant	c.4021delT	p.Ser1341fs	0.002	0	100	1	517
13	32,912,514	C	A	rs1135401901	*BRCA2*	stop gained	c.4022C>A	p.Ser1341*	0.003	0	100	1	517
13	32,912,557	C	CT	.	*BRCA2*	frameshift variant	c.4067dupT	p.Leu1356fs	0.002	0	100	1	403
13	32,912,655	CTTTT	C	.	*BRCA2*	frameshift variant	c.4166_4169delTTTT	p.Phe1389fs	0.002	0	100	1	502
13	32,912,751	ATTTTGAGACTTCTGATACAT	A	.	*BRCA2*	frameshift variant	c.4264_4283delGAGACTTCTGATACATTTTT	p.Glu1422fs	0.002	0	100	1	2,214
13	32,912,770	A	AT	rs1005805156; rs80359439	*BRCA2*	frameshift variant	c.4284dupT	p.Gln1429fs	0	0.003	100	1	2,116

© 2022 Momozawa Y et al. *JAMA Oncology.*

13	32,912,776	T	TC	.	*BRCA2*	frameshift variant	c.4285dupC	p.Gln1429fs	0.002	0	100	1	2,116
13	32,912,830	TG	T	rs80359443	*BRCA2*	frameshift variant	c.4339delG	p.Val1447fs	0.006	0.003	100	1	1,131
13	32,912,952	AAC	A	rs1064797176; rs397507720	*BRCA2*	frameshift variant	c.4464_4465delCA	p.His1488fs	0.002	0	100	1	1,484
13	32,912,961	TACTG	T	rs1442953012; rs80359451	*BRCA2*	frameshift variant	c.4471_4474delCTGA	p.Leu1491fs	0.002	0	100	1	1,484
13	32,912,964	TGAAA	T	rs1380446764; rs80359454	*BRCA2*	frameshift variant	c.4478_4481delAAAG	p.Glu1493fs	0	0.003	100	1	1,484
13	32,913,029	GATGA	G	.	*BRCA2*	frameshift variant	c.4539_4542delTGAA	p.Asp1513fs	0.003	0	100	1	1,902
13	32,913,138	AAG	A	.	*BRCA2*	frameshift variant	c.4649_4650delAG	p.Glu1550fs	0.005	0.003	100	1	2,318
13	32,913,211	T	TA	rs879255453	*BRCA2*	frameshift variant	c.4722dupA	p.Asp1575fs	0.002	0	100	1	1,994
13	32,913,261	AGT	A	.	*BRCA2*	frameshift variant	c.4772_4773delGT	p.Cys1591fs	0.002	0	99.999	1	834
13	32,913,295	T	TA	rs80359466	*BRCA2*	frameshift variant	c.4808dupA	p.Asn1603fs	0	0.003	99.999	1	834
13	32,913,314	G	T	.	*BRCA2*	stop gained	c.4822G>T	p.Glu1608*	0.005	0.003	99.999	1	1,669
13	32,913,359	C	T	.	*BRCA2*	stop gained	c.4867C>T	p.Gln1623*	0	0.003	100	1	1,741
13	32,913,391	CTT	C	.	*BRCA2*	frameshift variant	c.4903_4904delTT	p.Leu1635fs	0.005	0	100	1	1,741
13	32,913,435	CA	C	.	*BRCA2*	frameshift variant	c.4948delA	p.Ser1650fs	0.002	0	100	1	908
13	32,913,442	TC	T	rs397507752	*BRCA2*	frameshift variant	c.4952delC	p.Pro1651fs	0	0.005	100	1	908
13	32,913,457	C	A	rs80358721	*BRCA2*	stop gained	c.4965C>A	p.Tyr1655*	0.002	0	100	1	1,820
13	32,913,558	C	CA	rs1265028174; rs80359480	*BRCA2*	frameshift variant	c.5073dupA	p.Trp1692fs	0.003	0.003	100	1	1,375

© 2022 Momozawa Y et al. *JAMA Oncology.*

13	32,913,571	TAGAG	T	.	*BRCA2*	frameshift variant	c.5081_5084delGAGA	p.Arg1694fs	0.003	0	100	1	1,375
13	32,913,597	CAGAA	C	rs1362791447; rs879254123	*BRCA2*	frameshift variant	c.5110_5113delAGAA	p.Arg1704fs	0.002	0	100	1	461
13	32,913,619	TTATG	T	rs1209032259; rs80359484	*BRCA2*	frameshift variant	c.5130_5133delTGTA	p.Tyr1710fs	0.003	0	100	1	461
13	32,913,763	T	A	.	*BRCA2*	stop gained	c.5271T>A	p.Tyr1757*	0.002	0	100	1	1,509
13	32,913,771	C	A	.	*BRCA2*	stop gained	c.5279C>A	p.Ser1760*	0.002	0	100	1	1,509
13	32,913,844	C	CA	rs886040590	*BRCA2*	frameshift variant	c.5353dupA	p.Thr1785fs	0.002	0	100	1	2,097
13	32,913,900	CTG	C	rs80359512	*BRCA2*	frameshift variant	c.5410_5411delGT	p.Val1804fs	0.002	0	100	1	1,050
13	32,913,919	C	A	.	*BRCA2*	stop gained	c.5427C>A	p.Cys1809*	0.002	0	100	1	1,421
13	32,913,970	CATTAA	C	rs80359516	*BRCA2*	frameshift variant	c.5482_5486delAAATT	p.Lys1828fs	0.014	0.005	99.999	1	371
13	32,914,048	TTG	T	rs397507787	*BRCA2*	frameshift variant	c.5560_5561delGT	p.Val1854fs	0.003	0	99.999	1	371
13	32,914,065	CAATT	C	rs1255151416; rs80359520	*BRCA2*	frameshift variant	c.5576_5579delTTAA	p.Ile1859fs	0.136	0.011	100	1	623
13	32,914,087	A	AT	.	*BRCA2*	frameshift variant	c.5598dupT	p.Thr1867fs	0.003	0	100	1	624
13	32,914,127	G	T	rs55996097	*BRCA2*	stop gained	c.5635G>T	p.Glu1879*	0	0.003	100	1	624
13	32,914,137	C	A	rs80358785	*BRCA2*	stop gained	c.5645C>A	p.Ser1882*	0.030	0.011	100	1	624
13	32,914,137	CA	C	.	*BRCA2*	frameshift variant	c.5650delA	p.Ile1884fs	0.002	0	100	1	624
13	32,914,165	AG	A	.	*BRCA2*	frameshift variant	c.5675delG	p.Gly1892fs	0.003	0	100	1	253
13	32,914,174	C	G	rs41293497	*BRCA2*	stop gained	c.5682C>G	p.Tyr1894*	0.005	0	100	1	506

© 2022 Momozawa Y et al. *JAMA Oncology.*

13	32,914,199	AT	A	.	*BRCA2*	frameshift variant	c.5709delT	p.Leu1904fs	0.002	0	100	1	253
13	32,914,209	ACT	A	rs80359530	*BRCA2*	frameshift variant	c.5722_5723delCT	p.Leu1908fs	0.008	0.008	100	1	253
13	32,914,260	ACATT	A	rs80359535	*BRCA2*	frameshift variant	c.5771_5774delTTCA	p.Ile1924fs	0.003	0	100	1	660
13	32,914,264	TCA	T	.	*BRCA2*	frameshift variant	c.5773_5774delCA	p.Gln1925fs	0.003	0	100	1	660
13	32,914,472	C	T	rs80358831	*BRCA2*	stop gained	c.5980C>T	p.Gln1994*	0.002	0	100	1	1,057
13	32,914,503	AAGAT	A	rs1057518635	*BRCA2*	frameshift variant	c.6014_6017delATAG	p.Asp2005fs	0.005	0	100	1	1,057
13	32,914,552	ACATT	A	.	*BRCA2*	frameshift variant	c.6063_6066delTTCA	p.His2021fs	0.005	0	100	1	1,310
13	32,914,577	G	T	rs397507828	*BRCA2*	stop gained	c.6085G>T	p.Glu2029*	0.002	0	100	1	1,118
13	32,914,734	AG	A	.	*BRCA2*	frameshift variant	c.6244delG	p.Glu2082fs	0.002	0	100	1	466
13	32,914,736	G	T	rs886040642	*BRCA2*	stop gained	c.6244G>T	p.Glu2082*	0.002	0	100	1	466
13	32,914,790	CA	C	rs397507839	*BRCA2*	frameshift variant	c.6302delA	p.Asn2101fs	0.002	0	100	1	1,024
13	32,914,817	GT	G	.	*BRCA2*	frameshift variant	c.6327delT	p.Asp2110fs	0.003	0	100	1	559
13	32,914,839	AC	A	.	*BRCA2*	frameshift variant	c.6348delC	p.Cys2117fs	0.005	0	100	1	558
13	32,914,851	C	A	.	*BRCA2*	stop gained	c.6359C>A	p.Ser2120*	0.002	0	100	1	558
13	32,914,859	G	GA	rs80359577	*BRCA2*	frameshift variant	c.6373dupA	p.Thr2125fs	0.002	0	100	1	558
13	32,914,893	ATAACT	A	rs80359584	*BRCA2*	frameshift variant	c.6405_6409delCTTAA	p.Asn2135fs	0.028	0.013	100	1	559
13	32,914,897	CTTAAATG	C	rs397507851	*BRCA2*	frameshift variant	c.6408_6414delAAATGTT	p.Asn2137fs	0.002	0	100	1	559
13	32,914,906	TG	T	.	*BRCA2*	frameshift variant	c.6415delG	p.Glu2139fs	0.002	0	100	1	1,119
13	32,914,935	CTA	C	rs80359592	*BRCA2*	frameshift variant	c.6445_6446delAT	p.Ile2149fs	0.003	0	100	1	610

© 2022 Momozawa Y et al. *JAMA Oncology.*

13	32,914,936	TATTAA	T	rs80359593	*BRCA2*	frameshift variant	c.6446_6450delTTAAA	p.Ile2149fs	0.003	0	100	1	610
13	32,914,951	ATATCTCTCTCAAT	A	.	*BRCA2*	frameshift variant	c.6461_6473delATCTCTCTCAATT	p.Tyr2154fs	0.002	0	100	1	609
13	32,914,953	ATC	A	rs80359596	*BRCA2*	frameshift variant	c.6468_6469delTC	p.Gln2157fs	0.006	0	100	1	609
13	32,914,954	T	G	rs80358883	*BRCA2*	stop gained	c.6462T>G	p.Tyr2154*	0.005	0	100	1	609
13	32,914,963	AT	A	.	*BRCA2*	frameshift variant	c.6474delT	p.Gln2159fs	0.002	0	100	1	609
13	32,914,973	GACAA	G	rs1232139577; rs80359598	*BRCA2*	frameshift variant	c.6486_6489delACAA	p.Lys2162fs	0.002	0	100	1	610
13	32,915,007	CA	C	.	*BRCA2*	frameshift variant	c.6516delA	p.Val2174fs	0.002	0	100	1	996
13	32,915,043	AG	A	rs80359603	*BRCA2*	frameshift variant	c.6553delG	p.Ala2185fs	0.005	0.003	100	1	876
13	32,915,114	AAT	A	rs80359610	*BRCA2*	frameshift variant	c.6626_6627delTA	p.Ile2209fs	0.002	0	100	1	598
13	32,915,141	A	T	.	*BRCA2*	stop gained	c.6649A>T	p.Lys2217*	0.005	0	100	1	598
13	32,915,148	C	A	.	*BRCA2*	stop gained	c.6656C>A	p.Ser2219*	0.003	0	100	1	598
13	32,915,148	C	G	rs80358893	*BRCA2*	stop gained	c.6656C>G	p.Ser2219*	0.002	0	100	1	598
13	32,915,188	AG	A	.	*BRCA2*	frameshift variant	c.6697delG	p.Ala2233fs	0.002	0	99.957	1	158
13	32,915,212	GAC	G	.	*BRCA2*	frameshift variant	c.6722_6723delCA	p.Thr2241fs	0.002	0	99.96	1	158
13	32,915,292	C	G	.	*BRCA2*	stop gained	c.6800C>G	p.Ser2267*	0.002	0	99.961	1	161
13	32,918,775	A	T	.	*BRCA2*	stop gained	c.6922A>T	p.Lys2308*	0.008	0.003	99.152	1	240
13	32,918,778	AG	A	.	*BRCA2*	frameshift variant	c.6926delG	p.Ser2309fs	0.002	0	99.154	1	240

© 2022 Momozawa Y et al. *JAMA Oncology.*

13	32,920,962	A	G	rs81002863	*BRCA2*	splice acceptor variant & intron variant	c.6938-2A>G		0.002	0	100	1	8,822
13	32,920,978	C	T	rs80358920	*BRCA2*	stop gained	c.6952C>T	p.Arg2318*	0.169	0.027	100	1	8,822
13	32,929,050	C	T	rs80358936	*BRCA2*	stop gained	c.7060C>T	p.Gln2354*	0.002	0	100	1	2,131
13	32,929,143	G	GT	rs80359639	*BRCA2*	frameshift variant	c.7156dupT	p.Ser2386fs	0.002	0	100	1	1,557
13	32,929,199	CAA	C	rs80359642	*BRCA2*	frameshift variant	c.7211_7212delAA	p.Lys2404fs	0.002	0	100	1	507
13	32,929,299	AT	A	.	*BRCA2*	frameshift variant	c.7311delT	p.Ile2437fs	0.002	0	100	1	1,165
13	32,929,420	C	CT	rs779007406; rs886038168	*BRCA2*	frameshift variant & splice region variant	c.7433dupT	p.Leu2478fs	0.002	0	100	1	688
13	32,930,563	A	T	rs397507917	*BRCA2*	splice acceptor variant & intron variant	c.7436-2A>T		0.002	0	100	1	998
13	32,930,564	G	T	.	*BRCA2*	splice acceptor variant & intron variant	c.7436-1G>T		0.002	0	100	1	998
13	32,930,609	C	T	rs80358972	*BRCA2*	stop gained	c.7480C>T	p.Arg2494*	0	0.003	100	1	1,994
13	32,930,687	C	T	rs80358981	*BRCA2*	stop gained	c.7558C>T	p.Arg2520*	0.009	0	100	1	1,578
13	32,930,713	AG	A	.	*BRCA2*	frameshift variant	c.7586delG	p.Gly2529fs	0.002	0	100	1	2,158
13	32,930,744	C	T	rs886040720	*BRCA2*	stop gained & splice region variant	c.7615C>T	p.Gln2539*	0.002	0	100	1	1,164
13	32,931,931	CAG	C	rs80359672	*BRCA2*	frameshift variant	c.7673_7674delAG	p.Glu2558fs	0.002	0	100	1	807
13	32,931,963	T	TG	.	*BRCA2*	frameshift variant	c.7702_7703insG	p.Phe2568fs	0.002	0	100	1	798
13	32,931,976	GTT	G	.	*BRCA2*	frameshift variant	c.7717_7718delTT	p.Leu2573fs	0.002	0	100	1	798

© 2022 Momozawa Y et al. *JAMA Oncology.*

13	32,931,999	C	T	rs80358999	*BRCA2*	stop gained	c.7738C>T	p.Gln2580*	0.002	0	100	1	1,278
13	32,932,002	T	TTGGCTGA	.	*BRCA2*	frameshift variant & stop gained	c.7745_7751dupCTGATGG	p.Gly2585fs	0.002	0	100	1	962
13	32,932,053	G	T	rs1135401919	*BRCA2*	stop gained	c.7792G>T	p.Glu2598*	0.002	0	100	1	481
13	32,936,659	G	T	rs81002860	*BRCA2*	splice acceptor variant & intron variant	c.7806-1G>T		0.003	0	99.999	1	281
13	32,936,730	TG	T	.	*BRCA2*	frameshift variant	c.7878delG	p.Trp2626fs	0.002	0	99.999	1	282
13	32,936,732	G	A	rs80359013	*BRCA2*	stop gained	c.7878G>A	p.Trp2626*	0.002	0	99.999	1	282
13	32,936,805	A	AG	.	*BRCA2*	frameshift variant	c.7954dupG	p.Val2652fs	0.002	0	99.999	1	281
13	32,936,823	A	T	.	*BRCA2*	stop gained	c.7969A>T	p.Lys2657*	0.003	0	99.999	1	281
13	32,936,831	G	T	.	*BRCA2*	splice donor variant & intron variant	c.7976+1G>T		0.002	0	99.999	1	281
13	32,937,362	A	G	rs397507954	*BRCA2*	missense variant	c.8023A>G	p.Ile2675Val	0.025	0.005	100	1	833
13	32,937,377	GAC	G	rs276174901	*BRCA2*	frameshift variant	c.8042_8043delCA	p.Thr2681fs	0.002	0	100	1	835
13	32,937,479	C	T	rs80359058	*BRCA2*	stop gained	c.8140C>T	p.Gln2714*	0	0.003	100	1	1,344
13	32,937,507	A	G	rs41293513	*BRCA2*	missense variant	c.8168A>G	p.Asp2723Gly	0.005	0	100	1	1,344
13	32,937,582	G	A	rs80359071	*BRCA2*	missense variant	c.8243G>A	p.Gly2748Asp	0.005	0	100	1	511
13	32,944,571	G	A	rs397507981	*BRCA2*	stop gained	c.8364G>A	p.Trp2788*	0.002	0	99.978	1	584
13	32,944,696	T	C	rs886040944	*BRCA2*	splice donor variant & intron variant	c.8487+2T>C		0.002	0	99.976	1	584
13	32,945,172	AG	A	.	*BRCA2*	frameshift variant	c.8569delG	p.Ala2857fs	0.002	0	98.796	1	69

© 2022 Momozawa Y et al. *JAMA Oncology.*

13	32,950,825	ATT	A	.	*BRCA2*	frameshift variant	c.8653_8654delTT	p.Leu2885fs	0.003	0	100	1	1,132
13	32,950,930	T	A	.	*BRCA2*	splice donor variant & intron variant	c.8754+2T>A		0.002	0	100	1	566
13	32,953,544	AGGAAGGCCAT	A	.	*BRCA2*	frameshift variant	c.8850_8859delGGCCATGGAA	p.Lys2950fs	0.002	0	100	1	1,868
13	32,953,632	C	G	rs80359144	*BRCA2*	stop gained	c.8933C>G	p.Ser2978*	0.002	0	100	1	630
13	32,953,653	G	T	rs81002882	*BRCA2*	splice donor variant & intron variant	c.8953+1G>T		0.002	0	100	1	652
13	32,953,885	A	G	.	*BRCA2*	splice acceptor variant & intron variant	c.8954-2A>G		0.002	0	99.999	1	226
13	32,953,897	TA	T	rs886040807	*BRCA2*	frameshift variant	c.8965delA	p.Ile2989fs	0.002	0	99.999	1	226
13	32,953,985	AG	A	.	*BRCA2*	frameshift variant	c.9053delG	p.Ser3018fs	0.002	0	99.999	1	456
13	32,954,009	C	T	rs80359159	*BRCA2*	stop gained	c.9076C>T	p.Gln3026*	0.039	0.008	99.999	1	230
13	32,954,022	C	CA	rs1305653361	*BRCA2*	frameshift variant	c.9097dupA	p.Thr3033fs	0.008	0	99.999	1	230
13	32,954,042	C	T	rs397508037	*BRCA2*	stop gained	c.9109C>T	p.Gln3037*	0.005	0	99.999	1	231
13	32,954,050	G	A	rs28897756	*BRCA2*	splice region variant & synonymous variant	c.9117G>A	p.Pro3039Pro	0.016	0.003	99.999	1	231
13	32,954,272	G	GA	rs80359752; rs886038189	*BRCA2*	frameshift variant & splice region variant	c.9253dupA	p.Thr3085fs	0.002	0	99.998	1	371
13	32,968,951	C	T	rs80359212	*BRCA2*	stop gained	c.9382C>T	p.Arg3128*	0.009	0.003	100	1	1,463
13	32,971,153	T	TC	.	*BRCA2*	frameshift variant	c.9621dupC	p.Ile3208fs	0.002	0	100	1	358

© 2022 Momozawa Y et al. *JAMA Oncology.*

13	32,971,182	G	T	.	*BRCA2*	splice donor variant & intron variant	c.9648+1G>T		0.002	0.003	100	1	358
17	41,197,729	T	C	rs80357258	*BRCA1*	missense variant	c.5621A>G	p.Tyr1874Cys	0.011	0	100	1	1,159
17	41,197,766	T	TG	.	*BRCA1*	frameshift variant	c.5583dupC	p.Ser1862fs	0.002	0	100	1	2,317
17	41,197,784	G	A	rs41293465	*BRCA1*	stop gained	c.5566C>T	p.Arg1856*	0.003	0	100	1	2,318
17	41,199,659	C	T	rs80358145	*BRCA1*	splice donor variant & intron variant	c.5530+1G>A		0.003	0	100	1	1,564
17	41,201,183	AC	A	.	*BRCA1*	frameshift variant	c.5423delG	p.Cys1808fs	0.002	0	100	1	2,640
17	41,203,135	C	G	rs80358099	*BRCA1*	splice acceptor variant & intron variant	c.5341-1G>C		0.002	0.003	100	1	810
17	41,209,095	G	A	rs80357123	*BRCA1*	stop gained	c.5314C>T	p.Arg1772*	0.003	0	100	1	2,097
17	41,209,131	CT	C	.	*BRCA1*	frameshift variant	c.5277delA	p.Asp1760fs	0.002	0	100	1	2,095
17	41,209,133	CCT	C	.	*BRCA1*	frameshift variant	c.5274_5275delAG	p.Gly1759fs	0.002	0	100	1	1,075
17	41,209,134	C	T	rs80356937	*BRCA1*	missense variant	c.5275G>A	p.Gly1759Arg	0.002	0	100	1	1,049
17	41,215,349	C	T	rs80358004	*BRCA1*	splice donor variant & intron variant	c.5256+1G>A		0.003	0	100	1	810
17	41,215,382	G	A	rs878854957	*BRCA1*	stop gained	c.5224C>T	p.Gln1742*	0.005	0.003	100	1	811
17	41,215,895	A	T	rs397509230	*BRCA1*	stop gained	c.5211T>A	p.Tyr1737*	0.002	0	100	1	615
17	41,215,920	G	T	rs28897696	*BRCA1*	missense variant	c.5186C>A	p.Ala1729Glu	0.002	0	100	1	615

© 2022 Momozawa Y et al. *JAMA Oncology.*

17	41,215,947	C	T	rs41293459	*BRCA1*	missense variant	c.5159G>A	p.Arg1720Gln	0.011	0.008	100	1	1,230
17	41,215,948	G	A	rs55770810	*BRCA1*	missense variant	c.5158C>T	p.Arg1720Trp	0.002	0	100	1	1,230
17	41,215,951	C	A	.	*BRCA1*	stop gained	c.5155G>T	p.Glu1719*	0.002	0	100	1	1,229
17	41,215,954	A	G	rs80356993	*BRCA1*	missense variant	c.5152T>C	p.Cys1718Arg	0.002	0	100	1	1,230
17	41,215,969	C	G	rs1800747	*BRCA1*	splice acceptor variant & intron variant	c.5138-1G>C		0.002	0	100	1	1,230
17	41,219,625	C	G	rs80187739	*BRCA1*	missense variant & splice region variant	c.5137G>C	p.Asp1713His	0.002	0	100	1	1,587
17	41,219,634	TAAC	T	rs80358344	*BRCA1*	conservative inframe deletion	c.5125_5127delGTT	p.Val1709del	0.002	0	100	1	1,587
17	41,219,663	AG	A	rs80357896	*BRCA1*	frameshift variant	c.5098delC	p.Leu1700fs	0.002	0	100	1	1,586
17	41,219,665	ATTAG	A	rs80357580	*BRCA1*	frameshift variant	c.5093_5096delCTAA	p.Thr1698fs	0	0.003	100	1	1,586
17	41,223,031	T	TG	.	*BRCA1*	frameshift variant	c.4962dupC	p.Arg1655fs	0.002	0	100	1	653
17	41,223,054	T	TTATA	.	*BRCA1*	frameshift variant & stop gained	c.4936_4939dupTATA	p.Asn1647fs	0.002	0	100	1	826
17	41,223,060	C	CT	.	*BRCA1*	frameshift variant	c.4933_4934insA	p.Gly1645fs	0.002	0	100	1	826
17	41,226,411	G	A	rs80356992	*BRCA1*	stop gained	c.4675C>T	p.Gln1559*	0.003	0	100	1	1,202
17	41,226,540	T	C	rs80358054	*BRCA1*	splice acceptor variant & intron variant	c.4548-2A>G		0.005	0	100	1	1,202

© 2022 Momozawa Y et al. *JAMA Oncology.*

17	41,228,515	CTGGT	C	.	*BRCA1*	frameshift variant	c.4533_4536delACCA	p.Pro1512fs	0.002	0	100	1	616
17	41,228,557	C	CA	.	*BRCA1*	frameshift variant	c.4494dupT	p.Glu1499fs	0.002	0	100	1	1,233
17	41,234,439	G	GTTCT	rs397509164	*BRCA1*	frameshift variant	c.4335_4338dupAGAA	p.Gln1447fs	0.013	0.003	99.999	1	321
17	41,234,451	G	A	rs41293455	*BRCA1*	stop gained	c.4327C>T	p.Arg1443*	0.003	0	99.999	1	321
17	41,234,523	C	A	rs80357309	*BRCA1*	stop gained	c.4255G>T	p.Glu1419*	0.002	0	99.999	1	321
17	41,234,577	G	A	rs397509151	*BRCA1*	stop gained	c.4201C>T	p.Gln1401*	0.002	0	99.999	1	321
17	41,242,960	C	T	rs80358076	*BRCA1*	splice donor variant & intron variant	c.4185+1G>A		0.002	0	100	1	1,023
17	41,242,961	C	T	rs80356857	*BRCA1*	splice region variant & synonymous variant	c.4185G>A	p.Gln1395Gln	0.003	0	100	1	1,023
17	41,242,979	ACT	A	rs80357572	*BRCA1*	frameshift variant	c.4165_4166delAG	p.Ser1389fs	0.003	0	100	1	1,023
17	41,242,983	TGA	T	rs80357565	*BRCA1*	frameshift variant	c.4161_4162delTC	p.Gln1388fs	0.002	0	100	1	1,023
17	41,243,008	CAG	C	rs397509141	*BRCA1*	frameshift variant	c.4136_4137delCT	p.Ser1379fs	0.002	0	100	1	1,023
17	41,243,024	ACT	A	rs80357787	*BRCA1*	frameshift variant	c.4120_4121delAG	p.Ser1374fs	0.006	0	100	1	1,024
17	41,243,470	TTTGCTCTTCTTGATTATTTTCTTCC	T	.	*BRCA1*	frameshift variant	c.4053_4077delGGAAGAAAATAATCAAGAAGAGCAA	p.Glu1352fs	0.002	0	100	1	982
17	41,243,505	CCT	C	rs80357727	*BRCA1*	frameshift variant	c.4041_4042delAG	p.Gly1348fs	0.002	0	100	1	982

© 2022 Momozawa Y et al. *JAMA Oncology.*

17	41,243,539	C	CA	.	*BRCA1*	frameshift variant	c.4008dupT	p.Asp1337fs	0.002	0	100	1	983
17	41,243,569	G	A	rs876659720	*BRCA1*	stop gained	c.3979C>T	p.Gln1327*	0.002	0	100	1	2,850
17	41,243,615	GT	G	rs80357504	*BRCA1*	frameshift variant	c.3932delA	p.Asn1311fs	0.002	0	100	1	1,866
17	41,243,658	GA	G	rs886038027	*BRCA1*	frameshift variant	c.3889delT	p.Ser1297fs	0.002	0	100	1	1,868
17	41,243,707	G	A	rs80356866	*BRCA1*	stop gained	c.3841C>T	p.Gln1281*	0.002	0	100	1	921
17	41,243,776	CCT	C	rs1369534394; rs80357579	*BRCA1*	frameshift variant	c.3770_3771delAG	p.Glu1257fs	0.002	0	100	1	920
17	41,243,882	CT	C	.	*BRCA1*	frameshift variant	c.3665delA	p.Glu1222fs	0.002	0	100	1	966
17	41,243,901	A	C	rs397509091	*BRCA1*	stop gained	c.3647T>G	p.Leu1216*	0.003	0	100	1	966
17	41,243,908	C	A	rs80356923	*BRCA1*	stop gained	c.3640G>T	p.Glu1214*	0.008	0	100	1	965
17	41,243,941	G	A	rs62625308	*BRCA1*	stop gained	c.3607C>T	p.Arg1203*	0.002	0	100	1	488
17	41,243,967	G	GTGAA	.	*BRCA1*	frameshift variant	c.3577_3580dupTTCA	p.Thr1194fs	0.002	0	100	1	798
17	41,244,004	G	A	rs80357296	*BRCA1*	stop gained	c.3544C>T	p.Gln1182*	0.003	0.003	99.997	1	312
17	41,244,038	AATGTC	A	rs397509078	*BRCA1*	frameshift variant	c.3505_3509delGACAT	p.Asp1169fs	0.002	0	99.998	1	312
17	41,244,085	C	CT	rs80357857	*BRCA1*	frameshift variant	c.3462dupA	p.Asp1155fs	0.002	0	99.997	1	303
17	41,244,105	TC	T	rs80357808	*BRCA1*	frameshift variant	c.3442delG	p.Glu1148fs	0.011	0	99.996	1	302
17	41,244,145	G	A	rs80357136	*BRCA1*	stop gained	c.3403C>T	p.Gln1135*	0.002	0	100	1	1,506
17	41,244,149	TAA	T	rs80357577	*BRCA1*	frameshift variant	c.3397_3398delTT	p.Leu1133fs	0.003	0	100	1	1,506
17	41,244,159	GA	G	rs886040123	*BRCA1*	frameshift variant	c.3388delT	p.Ser1130fs	0.002	0	100	1	1,506

© 2022 Momozawa Y et al. *JAMA Oncology.*

17	41,244,218	CT	C	rs397509056	*BRCA1*	frameshift variant	c.3329delA	p.Lys1110fs	0.002	0	100	1	2,428
17	41,244,250	C	A	.	*BRCA1*	stop gained	c.3298G>T	p.Gly1100*	0.002	0	100	1	1,221
17	41,244,258	CTT	C	rs80357686	*BRCA1*	frameshift variant	c.3288_3289delAA	p.Leu1098fs	0.002	0	100	1	1,221
17	41,244,291	A	C	rs80357006	*BRCA1*	stop gained	c.3257T>G	p.Leu1086*	0.005	0	100	1	2,070
17	41,244,318	CCT	C	rs80357635	*BRCA1*	frameshift variant	c.3228_3229delAG	p.Gly1077fs	0.002	0	100	1	850
17	41,244,426	G	C	rs397509035	*BRCA1*	stop gained	c.3122C>G	p.Ser1041*	0.002	0	100	1	1,512
17	41,244,446	ATT	A	.	*BRCA1*	frameshift variant	c.3100_3101delAA	p.Asn1034fs	0.002	0	100	1	1,512
17	41,244,683	TGATAG	T	.	*BRCA1*	frameshift variant	c.2860_2864delCTATC	p.Leu954fs	0.002	0.003	100	1	470
17	41,244,687	AG	A	.	*BRCA1*	frameshift variant	c.2860delC	p.Leu954fs	0.002	0	100	1	470
17	41,244,748	G	A	rs80357223	*BRCA1*	stop gained	c.2800C>T	p.Gln934*	0.036	0	100	1	471
17	41,244,777	TTAAC	T	rs80357661	*BRCA1*	frameshift variant	c.2767_2770delGTTA	p.Val923fs	0.003	0	100	1	1,730
17	41,244,796	TGAT	GA	.	*BRCA1*	frameshift variant & missense variant	c.2749_2752delATCAinsTC	p.Ile917fs	0.002	0	100	1	1,729
17	41,244,869	C	CT	rs886040060	*BRCA1*	frameshift variant	c.2678dupA	p.Lys894fs	0.002	0	100	1	1,730
17	41,244,935	CGG	C	rs80357962	*BRCA1*	frameshift variant	c.2611_2612delCC	p.Pro871fs	0.002	0	100	1	1,250
17	41,244,997	C	A	rs398122662	*BRCA1*	stop gained	c.2551G>T	p.Glu851*	0.002	0	100	1	2,284
17	41,245,034	GT	G	rs80357863	*BRCA1*	frameshift variant	c.2513delA	p.Asn838fs	0.003	0	100	1	1,034
17	41,245,157	TTC	T	rs80357695	*BRCA1*	frameshift variant	c.2389_2390delGA	p.Glu797fs	0.022	0.005	100	1	1,406
17	41,245,247	GC	G	.	*BRCA1*	frameshift variant	c.2300delG	p.Ser767fs	0.002	0	100	1	749

© 2022 Momozawa Y et al. *JAMA Oncology.*

17	41,245,278	AC	A	rs80357583	*BRCA1*	frameshift variant	c.2269delG	p.Val757fs	0.003	0	100	1	363
17	41,245,330	C	CT	rs80357802	*BRCA1*	frameshift variant	c.2217dupA	p.Val740fs	0.002	0	100	1	1,367
17	41,245,351	CTTCTT	C	rs397508946	*BRCA1*	frameshift variant	c.2192_2196delAAGAA	p.Lys731fs	0.002	0	100	1	1,367
17	41,245,390	C	A	rs80356875	*BRCA1*	stop gained	c.2158G>T	p.Glu720*	0.002	0	100	1	2,022
17	41,245,410	G	C	rs80357233	*BRCA1*	stop gained	c.2138C>G	p.Ser713*	0.002	0	100	1	2,023
17	41,245,433	TG	T	.	*BRCA1*	frameshift variant	c.2114delC	p.Ala705fs	0.002	0	100	1	2,023
17	41,245,445	CT	C	.	*BRCA1*	frameshift variant	c.2102delA	p.Lys701fs	0.002	0	100	1	2,023
17	41,245,471	CAT	C	rs397508936	*BRCA1*	frameshift variant	c.2075_2076delAT	p.His692fs	0.002	0	100	1	1,673
17	41,245,475	TC	T	.	*BRCA1*	frameshift variant	c.2072delG	p.Arg691fs	0.002	0	100	1	1,673
17	41,245,518	CCA	C	rs397508931	*BRCA1*	frameshift variant	c.2028_2029delTG	p.Gly677fs	0.002	0	100	1	655
17	41,245,586	C	CT	rs80357853	*BRCA1*	frameshift variant	c.1961dupA	p.Tyr655fs	0.002	0	100	1	1,338
17	41,245,586	CT	C	rs80357522	*BRCA1*	frameshift variant	c.1961delA	p.Lys654fs	0.002	0	100	1	1,338
17	41,245,594	T	TC	rs80357753	*BRCA1*	frameshift variant	c.1953dupG	p.Lys652fs	0.002	0	100	1	685
17	41,245,716	AG	A	rs397508913	*BRCA1*	frameshift variant	c.1831delC	p.Leu611fs	0.002	0	99.986	1	221
17	41,245,735	CT	C	rs80357927	*BRCA1*	frameshift variant	c.1812delA	p.Ala605fs	0.002	0	100	1	441
17	41,246,029	TC	T	rs80357947	*BRCA1*	frameshift variant	c.1518delG	p.Arg507fs	0.003	0	99.999	1	556
17	41,246,029	T	A	rs397508880	*BRCA1*	stop gained	c.1519A>T	p.Arg507*	0.002	0	99.999	1	556
17	41,246,039	CTTTAA	C	rs80357888	*BRCA1*	frameshift variant	c.1504_1508delTTAAA	p.Leu502fs	0.002	0	99.999	1	556
17	41,246,068	G	A	rs80357010	*BRCA1*	stop gained	c.1480C>T	p.Gln494*	0.002	0	99.999	1	556

© 2022 Momozawa Y et al. *JAMA Oncology.*

17	41,246,101	TA	T	.	*BRCA1*	frameshift variant	c.1446delT	p.Ile483fs	0.002	0	99.999	1	557
17	41,246,169	AT	A	.	*BRCA1*	frameshift variant	c.1378delA	p.Ile460fs	0.002	0	99.999	1	556
17	41,246,255	TAAGTCTATTTTCTCTG	T	.	*BRCA1*	frameshift variant	c.1277_1292delCAGAGAAAATAGACTT	p.Ser426fs	0.005	0	100	1	830
17	41,246,427	GT	G	.	*BRCA1*	frameshift variant	c.1120delA	p.Thr374fs	0.002	0	100	1	1,259
17	41,246,435	AG	A	rs397508837	*BRCA1*	frameshift variant	c.1112delC	p.Pro371fs	0.002	0	100	1	1,260
17	41,246,637	A	AATTC	.	*BRCA1*	frameshift variant & stop gained	c.907_910dupGAAT	p.Phe304fs	0.003	0	100	1	2,317
17	41,246,742	A	T	.	*BRCA1*	stop gained	c.806T>A	p.Leu269*	0.002	0	99.999	1	359
17	41,247,917	G	A	rs397509301	*BRCA1*	stop gained	c.616C>T	p.Gln206*	0.003	0.003	100	1	2,164
17	41,251,834	G	A	rs80357133	*BRCA1*	stop gained	c.505C>T	p.Gln169*	0.003	0	100	1	782
17	41,251,881	CTG	C	rs80357882	*BRCA1*	frameshift variant	c.456_457delCA	p.Ser153fs	0.006	0.003	100	1	781
17	41,256,190	G	T	rs80356888	*BRCA1*	stop gained	c.390C>A	p.Tyr130*	0.003	0	100	1	469
17	41,256,236	GGA	G	rs80357881	*BRCA1*	frameshift variant	c.342_343delTC	p.Pro115fs	0.002	0	100	1	940
17	41,256,258	CA	C	rs80357544	*BRCA1*	frameshift variant	c.321delT	p.Phe107fs	0.002	0	100	1	480
17	41,256,280	T	G	rs80358011	*BRCA1*	splice acceptor variant & intron variant	c.302-2A>C		0.002	0	100	1	469
17	41,258,471	A	G	rs80358026	*BRCA1*	splice donor variant & intron variant	c.212+2T>C		0.002	0	100	1	576
17	41,258,495	A	G	rs80357064	*BRCA1*	missense variant	c.190T>C	p.Cys64Arg	0.002	0	100	1	576
17	41,258,497	A	T	rs80357086	*BRCA1*	stop gained	c.188T>A	p.Leu63*	0.122	0.022	100	1	576

© 2022 Momozawa Y et al. *JAMA Oncology.*

17	41,258,552	T	G	.	*BRCA1*	splice acceptor variant & intron variant	c.135-2A>C		0.003	0	100	1	1,153
17	41,267,741	A	C	rs80358131	*BRCA1*	splice donor variant & intron variant	c.134+2T>G		0.002	0	100	1	640
17	41,267,744	TGC	T	.	*BRCA1*	frameshift variant & splice region variant	c.131_132delGC	p.Cys44fs	0.003	0	100	1	640
17	41,267,797	C	T	rs80358018	*BRCA1*	splice acceptor variant & intron variant	c.81-1G>A		0.003	0	100	1	1,279
17	41,276,047	C	CT	rs1491544899; rs80357783	*BRCA1*	frameshift variant	c.66dupA	p.Glu23fs	0.002	0	100	1	1,481

Chromosome position is based on hg19. The effect of genetic variants on the amino acid sequence using the SnpEff ver4.3t. Protein position was reported according to CCDS11456 for *BRCA1* and CCDS9344 for *BRCA2*.

© 2022 Momozawa Y et al. *JAMA Oncology.*

eTable 2. Comparison of *BRCA1* and *BRCA2* pathogenic variant frequency for all* patients for each cancer type, versus controls.

	BRCA1			*BRCA2*		
	Carrier frequency (%)			Carrier frequency (%)		
**Cancer type**	**Case**	**Control**	**P value**	**Case**	**Control**	**P value**
Biliary tract	1.056	0.057	2.76×10^{-13}	1.056	0.175	1.58×10^{-6}
Breast (female)	1.271	0.040	3.40×10^{-17}	2.532	0.149	1.58×10^{-38}
Breast (male)	1.887	0.071	1.07×10^{-3}	18.868	0.199	8.04×10^{-31}
Cervical	0.263	0.040	5.23×10^{-3}	0.631	0.149	1.94×10^{-3}
Colorectal	0.159	0.057	4.96×10^{-4}	0.311	0.175	1.72×10^{-3}
Endometrial	0.261	0.040	1.31×10^{-3}	0.522	0.149	1.94×10^{-3}
Esophageal	0.323	0.057	3.59×10^{-5}	0.738	0.175	7.16×10^{-8}
Gastric	0.273	0.057	8.59×10^{-9}	0.894	0.175	5.29×10^{-25}
Liver	0.194	0.057	2.04×10^{-3}	0.443	0.175	8.72×10^{-4}
Lung	0.243	0.057	3.02×10^{-6}	0.567	0.175	1.83×10^{-8}
Lymphoma	0.354	0.057	8.82×10^{-6}	0.472	0.175	0.012
Ovarian	4.860	0.040	8.96×10^{-34}	3.422	0.149	9.46×10^{-38}
Pancreatic	0.697	0.057	2.43×10^{-9}	2.291	0.175	6.40×10^{-27}
Prostate	0.162	0.071	0.012	1.009	0.199	1.36×10^{-17}
Renal	0.132	0.057	0.402	0.527	0.175	0.031

*Patients with reported family history were included. See methods for more details.
A logistic regression analysis under a dominant model with age at diagnosis for cases and age at registration for controls as covariate was used. We eliminated samples without age diagnosis or registration from this calculation.

© 2022 Momozawa Y et al. *JAMA Oncology.*

eTable 3. Results of two sensitivity analyses in breast cancer.

Methods		*BRCA1*				*BRCA2*		
		OR	95 % CI	*P* value		OR	95 % CI	*P* value
1. Logistic regression analysis adjusting for region		23.0	9.7 - 54.8	1.35×10^{-12}		10.0	6.3 - 15.8	1.78×10^{-21}
2. Burden test in patients with breast cancer only		15.2	6.6 - 34.9	1.38×10^{-10}		10.7	6.8 - 16.8	9.23×10^{-23}

We conducted two sensitivity analyses in breast cancer to investigate a potential bias from population stratification and the presence of more than one cancer type. Both results were comparable to estimates from the main analysis shown in Table 2

© 2022 Momozawa Y et al. *JAMA Oncology.*

eTable 4. Mean age at diagnosis of each cancer type in patients with or without pathogenic variants in *BRCA1* and *BRCA2*.

	BRCA1				*BRCA2*			
**Cancer type**	**Carrier**	**Non-carrier**	**Difference**	**P value**	**Carrier**	**Non-carrier**	**Difference**	**P value**
Biliary tract	72.9 ± 9.4	68.7 ± 9.5	4.2	0.22	-	-	-	-
Breast (female)	50.7 ± 12.5	56.4 ± 12.1	-5.7	2.06×10^{-6}	50.7 ± 11.5	56.4 ± 12.1	-5.7	2.48×10^{-13}
Breast (male)	-	-	-	-	71.9 ± 9.6	67.0 ± 10.1	4.9	0.19
Esophageal	-	-	-	-	60.9 ± 7.7	65.2 ± 8.5	-4.3	0.04
Gastric	62.3 ± 12.0	65.7 ± 10.5	-3.5	0.14	64.5 ± 9.7	65.7 ± 10.5	-1.3	0.22
Ovarian	55.8 ± 10.1	53.5 ± 12.3	2.3	0.07	57.5 ± 9.8	53.5 ± 12.2	4.1	6.31×10^{-3}
Pancreatic	61.6 ± 8.2	67.2 ± 9.9	-5.7	0.12	67.4 ± 12.5	67.2 ± 9.9	0.2	0.94
Prostate	-	-	-	-	68.1 ± 8.0	70.3 ± 7.2	-2.2	5.79×10^{-3}

t-test was used for this comparison. We eliminated samples without age at diagnosis. P = 0.01 in *BRCA1* and P = 7.14×10^{-3} in *BRCA2* were set at the threshold of significance.

eTable 5. Comparisons of histological subtypes between carriers with pathogenic variants and non-carriers.

(A) Biliary tract cancer

	BRCA1			*BRCA2*		
**Histological type**	**Carrier (%)**	**Non-carrier (%)**	**P value**	**Carrier (%)**	**Non-carrier (%)**	**P value**
Papillary adenocarcinoma	16.7	11.9	0.60	-	-	NA
Tubular adenocarcinoma	83.3	73.9		-	-	
Others	0.0	14.2		-	-	

(B) Female breast cancer

	BRCA1			*BRCA2*		
**Histological type**	**Carrier (%)**	**Non-carrier (%)**	**P value**	**Carrier (%)**	**Non-carrier (%)**	**P value**
Invasive papillotubular carcinoma	29.7	40.1	1.19×10^{-6}	35.1	40.1	2.95×10^{-3}
Invasive solid-tubular carcinoma	30.7	12.6		18.1	12.7	
Invasive scirrhous carcinoma	25.7	27.2		33.7	27.0	
Others	13.9	20.1		13.2	20.2	

(C) Esophageal cancer

	BRCA1			*BRCA2*		
**Histological type**	**Carrier (%)**	**Non-carrier (%)**	**P value**	**Carrier (%)**	**Non-carrier (%)**	**P value**
Squamous cell carcinoma	-	-	NA	80.0	91.6	0.26
Others	-	-		20.0	8.4	

© 2022 Momozawa Y et al. *JAMA Oncology.*

**(D) Gastric cancer**							
	BRCA1				***BRCA2***		
**Histological type**	**Carrier (%)**	**Non-carrier (%)**	**P value**		**Carrier (%)**	**Non-carrier (%)**	**P value**
Tubular adenocarcinoma	73.1	66.7	0.62		65.9	66.7	0.16
Poorly differentiated adenocarcinoma	19.2	17.4			21.2	17.4	
Signet-ring cell carcinoma	7.7	10.7			4.7	10.8	
Others	0.0	5.2			8.2	5.1	
**(E) Ovarian cancer**							
	BRCA1				***BRCA2***		
**Histological type**	**Carrier (%)**	**Non-carrier (%)**	**P value**		**Carrier (%)**	**Non-carrier (%)**	**P value**
Serous adenocarcinoma	63.5	28.8	6.60×10^{-9}		73.3	28.9	1.75×10^{-8}
Clear cell adenocarcinoma	1.6	21.8			6.7	21.4	
Endometrioid adenocarcinoma	17.5	18.4			6.7	18.8	
Mucinous adenocarcinoma	1.6	16.3			2.2	16.1	
Others	15.9	14.7			11.1	14.9	
**(F) Pancreatic cancer**							
	BRCA1				***BRCA2***		
**Histological type**	**Carrier (%)**	**Non-carrier (%)**	**P value**		**Carrier (%)**	**Non-carrier (%)**	**P value**
Invasive ductal carcinoma	40.0	61.2	0.63		84.6	60.4	0.20
Intraductal papillary mucinous carcinoma	20.0	12.5			7.7	12.7	
Others	40.0	26.3			7.7	26.9	
**(G) Prostate cancer**							
	BRCA1				***BRCA2***		
**Histological type**	**Carrier (%)**	**Non-carrier (%)**	**P value**		**Carrier (%)**	**Non-carrier (%)**	**P value**
Adenocarcinoma	-	-	NA		100.0	99.5	1.00
Others	-	-			0.0	0.5	

P-value was calculated using the χ^2 test.

© 2022 Momozawa Y et al. *JAMA Oncology.*

5. Supplementary references

1. Spurdle AB, Healey S, Devereau A, et al. ENIGMA--evidence-based network for the interpretation of germline mutant alleles: an international initiative to evaluate risk and clinical significance associated with sequence variation in BRCA1 and BRCA2 genes. *Hum. Mutat.* 2012;33(1):2-7.

2. Cancer Registry and Statistics. Cancer Information Service, National Cancer Center, Japan (Ministry of Health, Labour and Welfare, National Cancer Registry).

3. Koyanagi YN, Ito H, Oze I, et al. Development of a prediction model and estimation of cumulative risk for upper aerodigestive tract cancer on the basis of the aldehyde dehydrogenase 2 genotype and alcohol consumption in a Japanese population. *Eur. J. Cancer Prev.* 2017;26(1):38-47.

4. Crispo A, Brennan P, Jockel KH, et al. The cumulative risk of lung cancer among current, ex- and never-smokers in European men. *Br. J. Cancer.* 2004;91(7):1280-1286.

5. Peto R, Darby S, Deo H, Silcocks P, Whitley E, Doll R. Smoking, smoking cessation, and lung cancer in the UK since 1950: combination of national statistics with two case-control studies. *BMJ.* 2000;321(7257):323-329.

6. Giordano SH. Breast Cancer in Men. *N. Engl. J. Med.* 2018;378(24):2311-2320.
